# Supplementary material for: An animal toxin-antidote system kills cells by creating a novel cation channel
Source: PLoS Biol. 2025 May 27;23(5):e3003182. doi: 10.1371/journal.pbio.3003182 (PMC12136403; doi:10.1371/journal.pbio.3003182)
Supplement: S1 Fig — (A) Genetic cross to test for paternal versus zygotic effect of sperm-delivered PEEL-1 suppression by pmpl-1(yak103) deletion mutant (denoted pmpl-1(−)). Males heterozygous for the selfish element and carrying pmpl-1(−) are mated to p(−) z(−); pmpl-1(+) hermaphrodites. Suppression via paternal effect would result in approximately 0% embryonic lethality while suppression by a zygotic effect would result in approximately 50% lethality of cross-progeny. (B) The percent of dead embryos seen from the cross shown in panel (A). Results are consistent with a model of pmpl-1(yak103) suppression of PEEL-1 through zygotic effect. Underlying data are available in S2 Data. (PDF) [file pbio.3003182.s001.pdf]

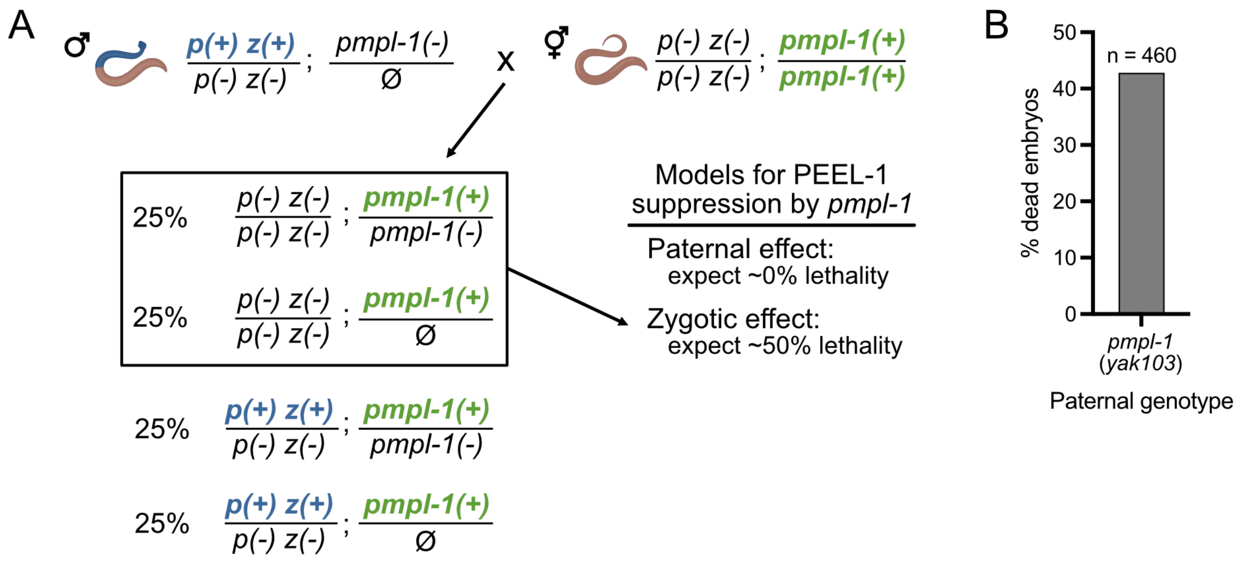

S1 Fig. *pmpl-1*(*yak103*) does not act through paternal-effect.

(A) Genetic cross to test for paternal versus zygotic effect of sperm-delivered PEEL-1 suppression by *pmpl-1(yak103)* deletion mutant (denoted *pmpl-1(-)*). Males heterozygous for the selfish element and carrying *pmpl-1(-)* are mated to *p(-) z(-); pmpl-1(+)* hermaphrodites. Suppression via paternal effect would result in ~0% embryonic lethality while suppression by a zygotic effect would result in ~50% lethality of cross-progeny. **(B)** The percent of dead embryos seen from the cross shown in panel (A). Results are consistent with a model of *pmpl-1*(*yak103*) suppression of PEEL-1 through zygotic effect. Underlying data are available in S2 Data.
