# Supplementary material for: An animal toxin-antidote system kills cells by creating a novel cation channel
Source: PLoS Biol. 2025 May 27;23(5):e3003182. doi: 10.1371/journal.pbio.3003182 (PMC12136403; doi:10.1371/journal.pbio.3003182)
Supplement: S2 Fig — (A) Maximum intensity projection of a C. elegans, 1.5-fold embryo with GFP driven by the pmpl-1 promoter and co-injection markers myo-2p::mCherry, rab-3p::mCherry, and myo-3p::mCherry. Toxicity from sperm-delivered PEEL-1 occurs after this embryonic stage. (B) Maximum intensity projections of hermaphrodite adult head (top), midbody (middle), and tail (bottom) of the same strain shown in panel (A). (C) Heatmap of pmpl-1 tissue expression scores from RNA-seq dataset of Day 1 adult worms from Kaletsky and colleagues, 2018 [6]. Tissues with the highest pmpl-1 expression (red) and lowest expression (blue) are shown. Only the 8 most highly expressed tissues (left) and the 8 most lowly expressed tissues (right) are shown. Expression of pmpl-1 is lowest in the male gonad (arrow). (D) pmpl-1(yak103) worms with vulval muscle cell expression of peel-1::GFP alone (top) or with pmpl-1::GFP (bottom). Number of scored worms are indicated. Green channel brightness is increased in the bottom panel to show fluorescence in the vulval muscle, since toxicity in this cell likely caused decreased levels of fluorescent-tagged proteins. The vulval muscle appears swollen in the green channel when co-expressing peel-1 and pmpl-1. This is the same worm as shown in Fig 1E. (PDF) [file pbio.3003182.s002.pdf]

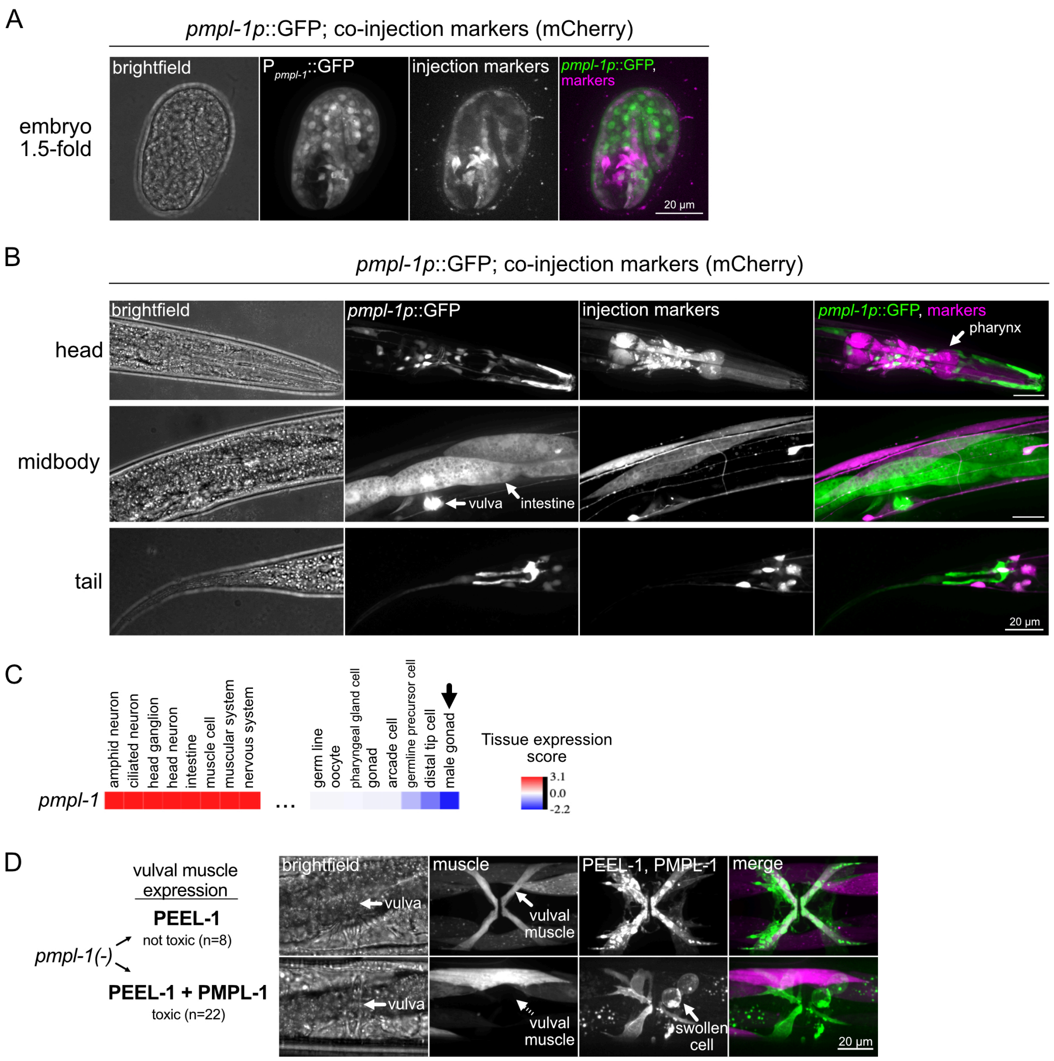

S2 Fig. *pmpl-1* expression pattern in *C. elegans*.

(A) Maximum intensity projection of a *C. elegans*, 1.5-fold embryo with GFP driven by the *pmpl-1* promoter and co-injection markers *myo-2p*::mCherry, *rab-3p*::mCherry, and *myo-3p*::mCherry. Toxicity from sperm-delivered PEEL-1 occurs after this embryonic stage. **(B)** Maximum intensity projections of hermaphrodite adult head (top), midbody (middle), and tail (bottom) of the same strain shown in panel (A). **(C)** Heatmap of *pmpl-1* of tissue expression scores from RNA-seq dataset of Day 1 adult worms from Kaletsky et al., 2018 [6]. Tissues with the highest *pmpl-1* expression (red) and lowest expression (blue) are shown. Only the 8 most highly expressed tissues (left) and the 8 most lowly expressed tissues (right) are shown. Expression of *pmpl-1* is lowest in the male gonad (arrow). **(D)** *pmpl-1(yak103)* worms with vulval muscle cell expression of *peel-1*::GFP alone (top) or with *pmpl*-1::GFP (bottom). Number of scored worms are indicated. Green channel brightness is increased in the bottom panel to show fluorescence in the vulval muscle, since toxicity in this cell likely caused decreased levels of fluorescent-tagged proteins. The vulval muscle appears swollen in the green channel when co-expressing *peel-1* and *pmpl-1*. This is the same worm as shown in Fig 1E.
