# Supplementary material for: An animal toxin-antidote system kills cells by creating a novel cation channel
Source: PLoS Biol. 2025 May 27;23(5):e3003182. doi: 10.1371/journal.pbio.3003182 (PMC12136403; doi:10.1371/journal.pbio.3003182)
Supplement: S3 Fig — Alignment of PMPL-1 amino acid sequences (right) between representative Caenorhabditis species, Pristionchus pacificus, and Toxocara canis shown in a species phylogeny (left). The pmpl-1 (yak52) allele is mutated at conserved residue A47 (*). (PDF) [file pbio.3003182.s003.pdf]

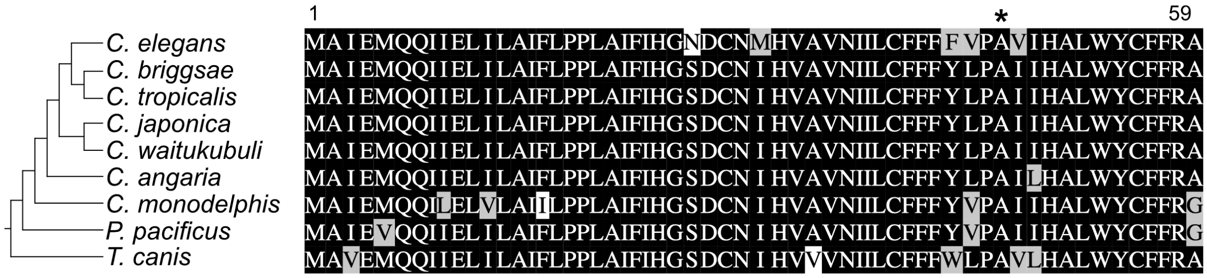

S3 Fig. PMPL-1 is conserved in nematodes.

Alignment of PMPL-1 amino acid sequences (right) between representative *Caenorhabditis* species, *Pristionchus pacificus*, and *Toxocara canis* shown in a species phylogeny (left). The *pmpl-1* (*yak52*) allele is mutated at conserved residue A47 (*).
