# Supplementary material for: An animal toxin-antidote system kills cells by creating a novel cation channel
Source: PLoS Biol. 2025 May 27;23(5):e3003182. doi: 10.1371/journal.pbio.3003182 (PMC12136403; doi:10.1371/journal.pbio.3003182)
Supplement: S4 Fig — Alignment of all 15 PMP3-like proteins in C. elegans identified by BLAST. (PDF) [file pbio.3003182.s004.pdf]

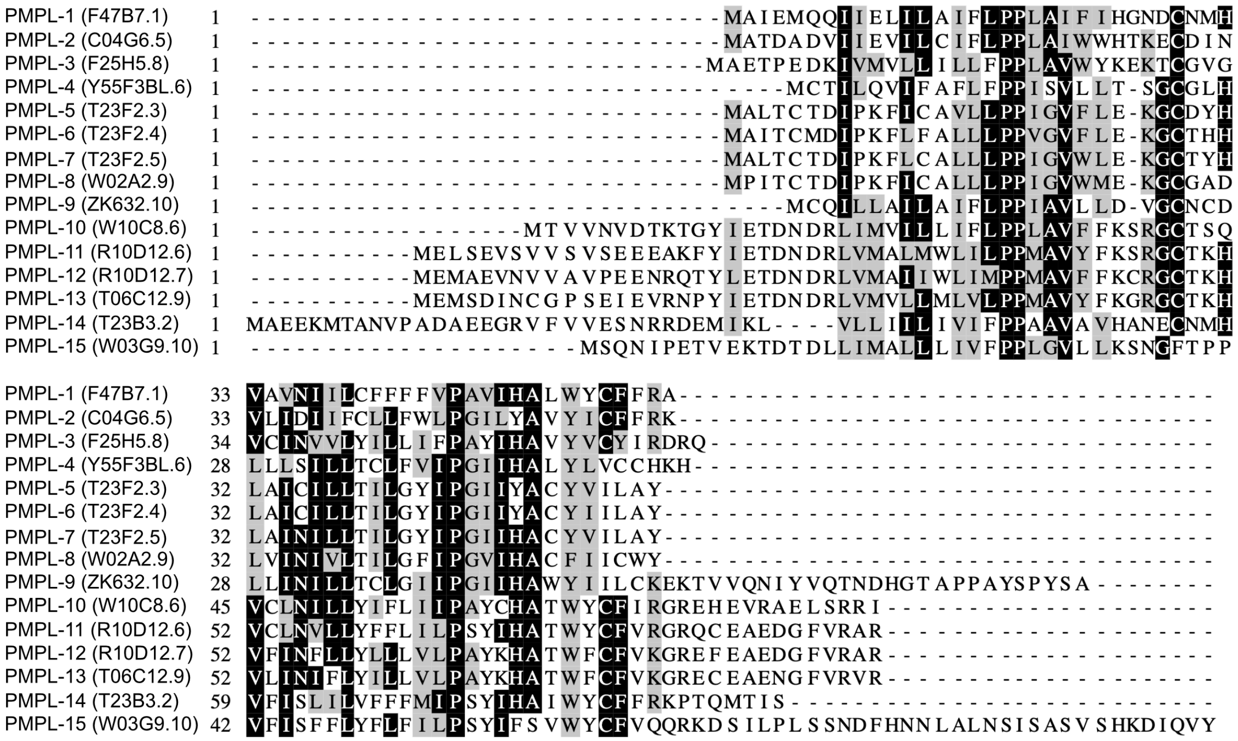

S4 Fig. 15 PMP3-like proteins in *C. elegans*.

Alignment of all 15 PMP3-like proteins in *C. elegans* identified by BLAST.
