# Supplementary material for: An animal toxin-antidote system kills cells by creating a novel cation channel
Source: PLoS Biol. 2025 May 27;23(5):e3003182. doi: 10.1371/journal.pbio.3003182 (PMC12136403; doi:10.1371/journal.pbio.3003182)
Supplement: S5 Fig — DeepTMHMM predictions of (A) PMPL-1 and (C) PEEL-1. PMPL-1 is predicted to be a two-pass transmembrane protein. PEEL-1 is predicted to be a four-pass transmembrane protein. Both proteins are predicted to have their N- and C-termini facing the cytosol. Structural predictions from AlphaFold2 of (B) PMPL-1 and (D) PEEL-1. AlphaFold2 predicts a very high-confidence PMPL-1 structure that suggests it is a monotopic protein (passing through one leaflet of a lipid bilayer). The PEEL-1 structure is of low and very-low confidence, likely because there are no known homologs of this protein. (PDF) [file pbio.3003182.s005.pdf]

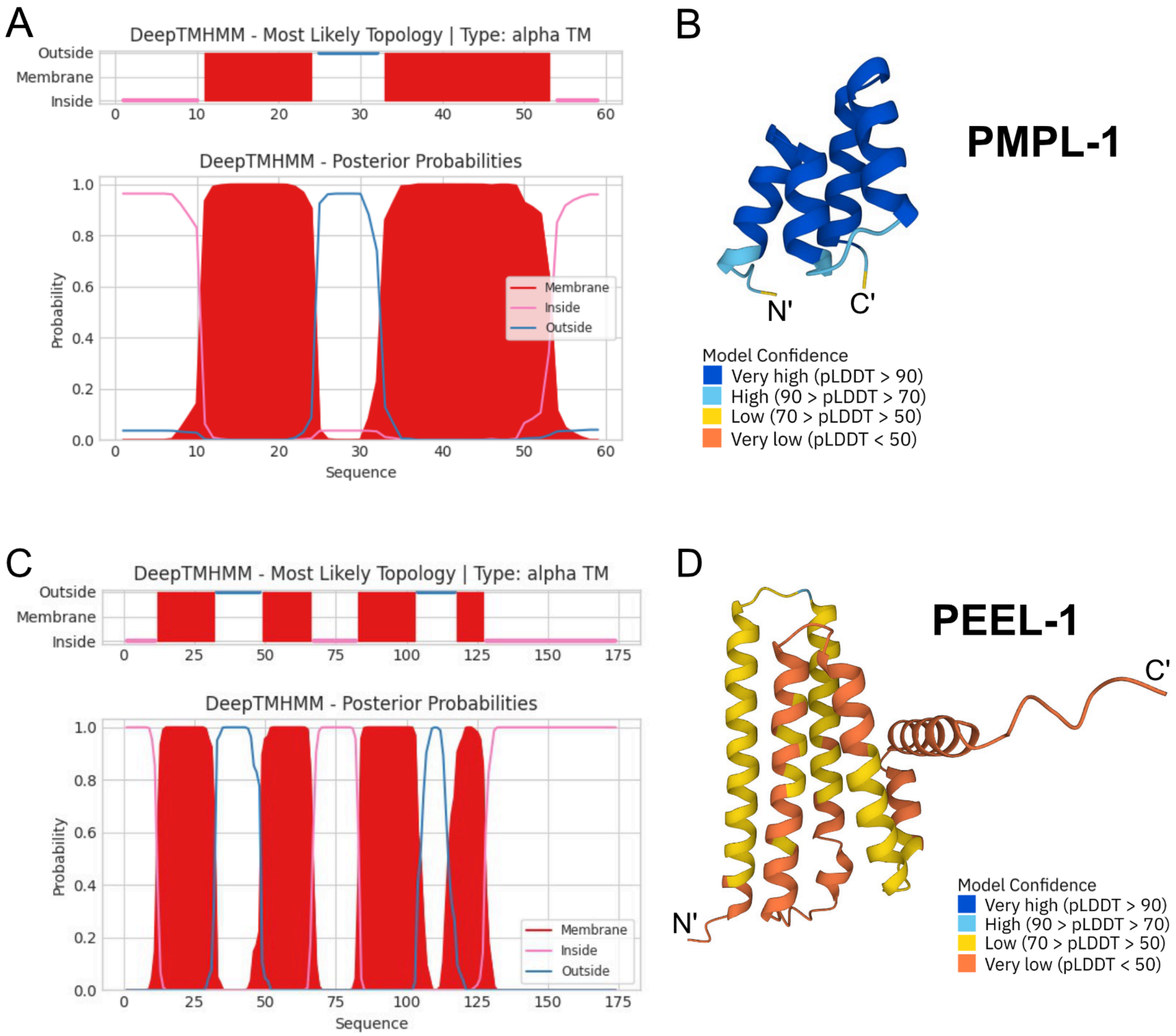

S5 Fig. Structural predictions of PEEL-1 and PMPL-1.

DeepTMHMM predictions of **(A)** PMPL-1 and **(C)** PEEL-1. PMPL-1 is predicted to be a two-pass transmembrane protein. PEEL-1 is predicted to be a four-pass transmembrane protein. Both proteins are predicted to have their N- and C-termini facing the cytosol. Structural predictions from AlphaFold2 of **(B)** PMPL-1 and **(D)** PEEL-1. AlphaFold2 predicts a very high-confidence PMPL-1 structure that suggests it is a monotopic protein (passing through one leaflet of a lipid bilayer). The PEEL-1 structure is of low and very-low confidence, likely because there are no known homologs of this protein.
