# Supplementary material for: An animal toxin-antidote system kills cells by creating a novel cation channel
Source: PLoS Biol. 2025 May 27;23(5):e3003182. doi: 10.1371/journal.pbio.3003182 (PMC12136403; doi:10.1371/journal.pbio.3003182)
Supplement: S6 Fig — (A–B) Intestinal cells of an adult C. elegans worm with the indicated constructs. (A) Wild-type worms expressing pmpl-1::tagRFP and GFP::dgat-2. DGAT-2 localizes to lipid droplet membranes, and PMPL-1::tagRFP co-localizes to these organelles. Inset shows one lipid droplet. (B) pmpl-1(yak103) expressing GFP::dgat-2 and peel-1::tagRFP. PEEL-1 signal (arrow) appears on plasma membrane lining the intestinal lumen and does not co-localize with lipid droplets. Autofluorescence from gut granules appears as filled-in circles in both channels (arrowhead). (C) HEK293 cells stably expressing peel-1::eGFP. PEEL-1::eGFP localizes to the ER and plasma membrane (arrows). Scale bar = 10 µm. (PDF) [file pbio.3003182.s006.pdf]

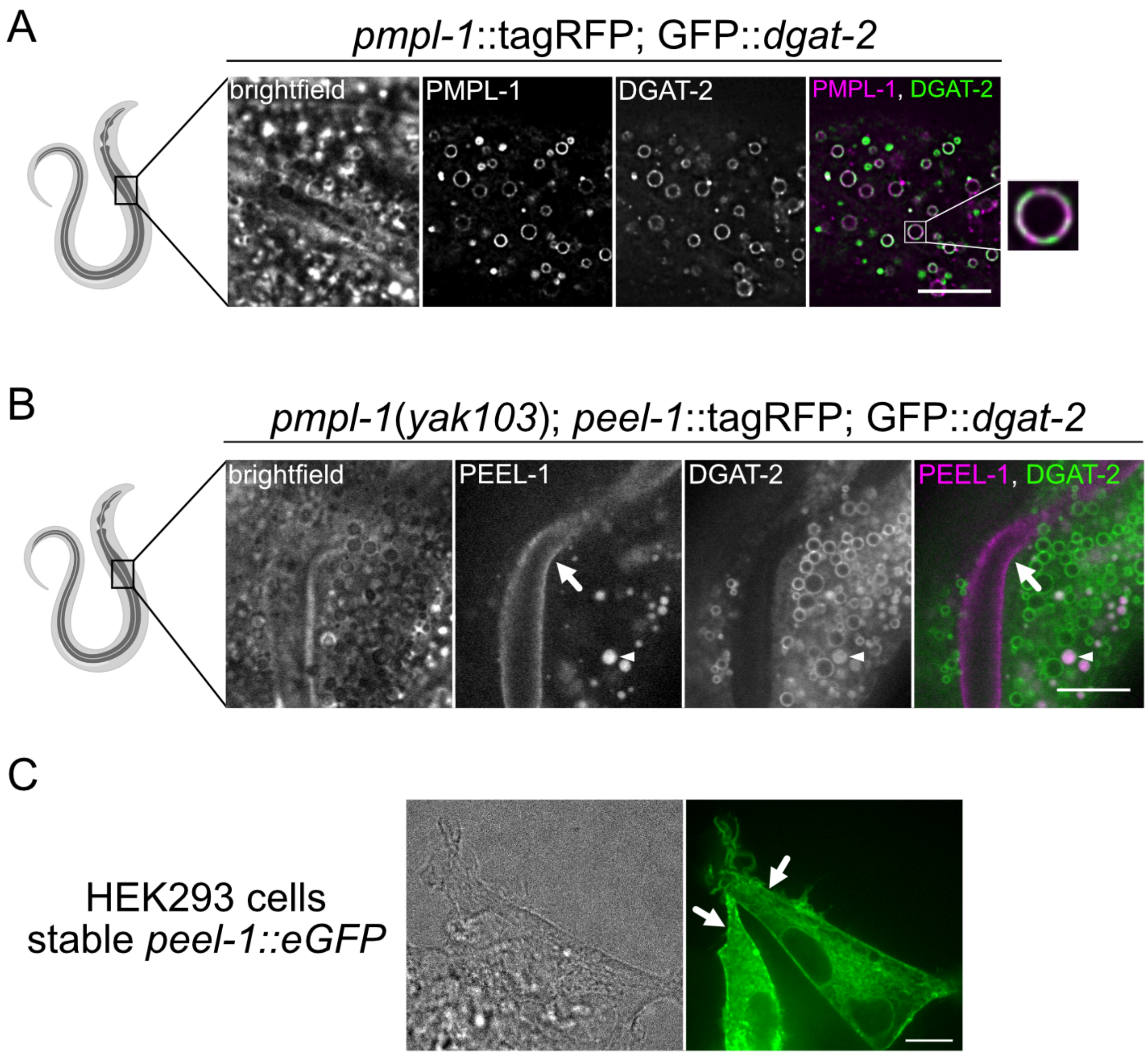

S6 Fig. PEEL-1 and PMPL-1 localization.

(A-B) Intestinal cells of an adult *C. elegans* worm with the indicated constructs. **(A)** Wild-type worms expressing *pmpl-1*::tagRFP and GFP::*dgat-2*. DGAT-2 localizes to lipid droplet membranes, and PMPL-1::tagRFP co-localizes to these organelles. Inset shows one lipid droplet. **(B)** *pmpl-1*(*yak103*) expressing GFP::*dgat-2* and *peel-1*::tagRFP. PEEL-1 signal (arrow) appears on plasma membrane lining the intestinal lumen and does not co-localize with lipid droplets. Autofluorescence from gut granules appears as filled-in circles in both channels (arrowhead). **(C)** HEK293 cells stably expressing *peel-1*::eGFP. PEEL-1::eGFP localizes to the ER and plasma membrane (arrows). Scale bar = 10μm.
