# Supplementary material for: An animal toxin-antidote system kills cells by creating a novel cation channel
Source: PLoS Biol. 2025 May 27;23(5):e3003182. doi: 10.1371/journal.pbio.3003182 (PMC12136403; doi:10.1371/journal.pbio.3003182)
Supplement: S7 Fig — Live-cell imaging time course of two HEK293T cells transfected with constructs coding for PEEL-1 (untagged), PMPL-1::eGFP, and mCherry::KDEL (ER marker). Imaging began at 20 hrs post-transfection (t = 0 min). The top cell experiences a loss of plasma membrane (PM) integrity at 25 min, followed by ER swelling. The lower cell loses PM integrity at 150 min. Scale bar = 10 µm. (PDF) [file pbio.3003182.s007.pdf]

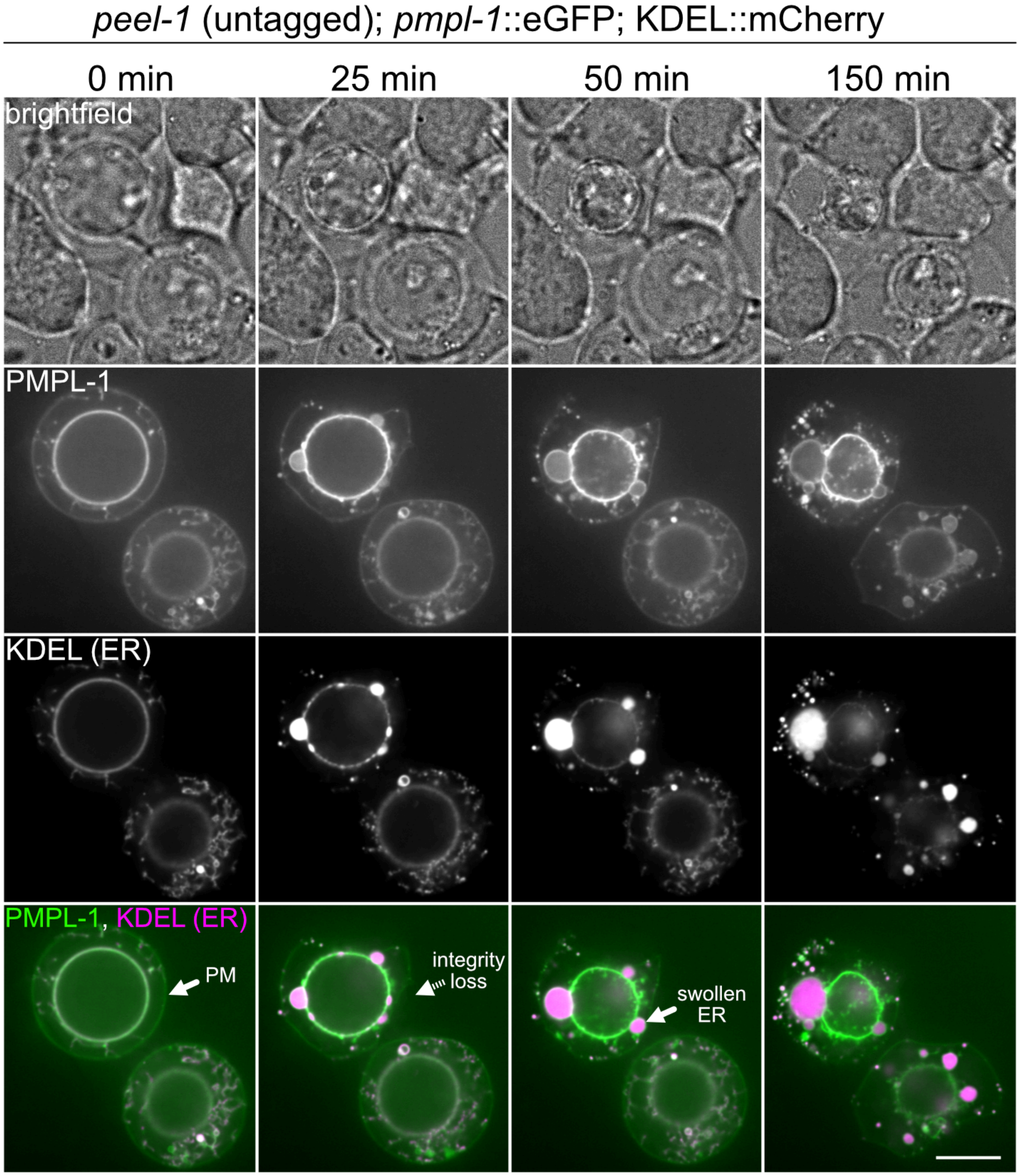

S7 Fig. Toxicity causes ER swelling, ER fragmentation, and cell lysis.

Live-cell imaging time course of two HEK293T cells transfected with constructs coding for PEEL-1 (untagged), PMPL-1::eGFP, and mCherry::KDEL (ER marker). Imaging began at 20 hours post-transfection (t=0 min). The top cell experiences a loss of plasma membrane (PM) integrity at 25 min, followed by ER swelling. The lower cell loses PM integrity at 150 min. Scale bar = 10μm.
