# Supplementary material for: An animal toxin-antidote system kills cells by creating a novel cation channel
Source: PLoS Biol. 2025 May 27;23(5):e3003182. doi: 10.1371/journal.pbio.3003182 (PMC12136403; doi:10.1371/journal.pbio.3003182)
Supplement: S8 Fig — Percent dead worms after heat-shock induced expression of PEEL-1. Worms deficient in apoptosis (ced-3) and cell engulfment (ced-2 and ced-5) were tested. Two independent experiments were done, with n = 50 worms for each data point. Underlying data are available in S2 Data. (PDF) [file pbio.3003182.s008.pdf]

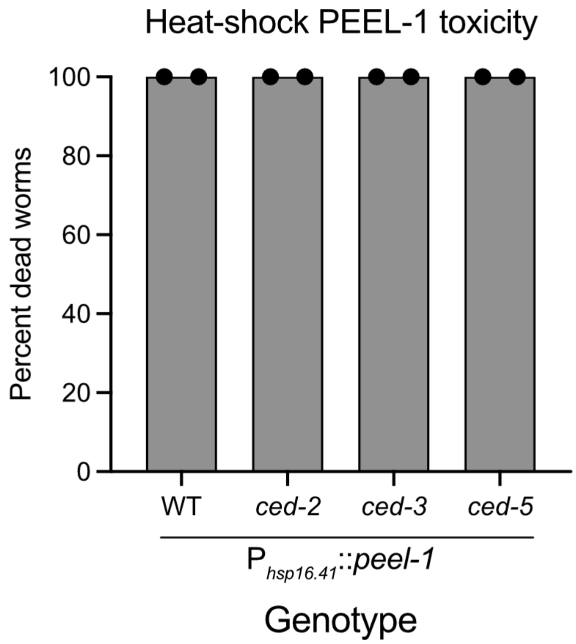

S8 Fig. Ectopic PEEL-1 toxicity is non-apoptotic.

Percent dead worms after heat-shock induced expression of PEEL-1. Worms deficient in apoptosis (*ced-3*) and cell engulfment (*ced-2* and *ced-5*) were tested. Two independent experiments were done, with n=50 worms for each data point. Underlying data are available in S2 Data.
