# Supplementary material for: An animal toxin-antidote system kills cells by creating a novel cation channel
Source: PLoS Biol. 2025 May 27;23(5):e3003182. doi: 10.1371/journal.pbio.3003182 (PMC12136403; doi:10.1371/journal.pbio.3003182)
Supplement: S9 Fig — Hydrophobic moment (µH) and hydrophobicity (H) of the amphipathic helix of 35 Actinoporin toxin proteins (data acquired from Macrander and Daly, 2016 [29]) (gray circles) and the predicted PEEL-1 amphipathic helix (blue square). Underlying data are available in S2 Data. (PDF) [file pbio.3003182.s009.pdf]

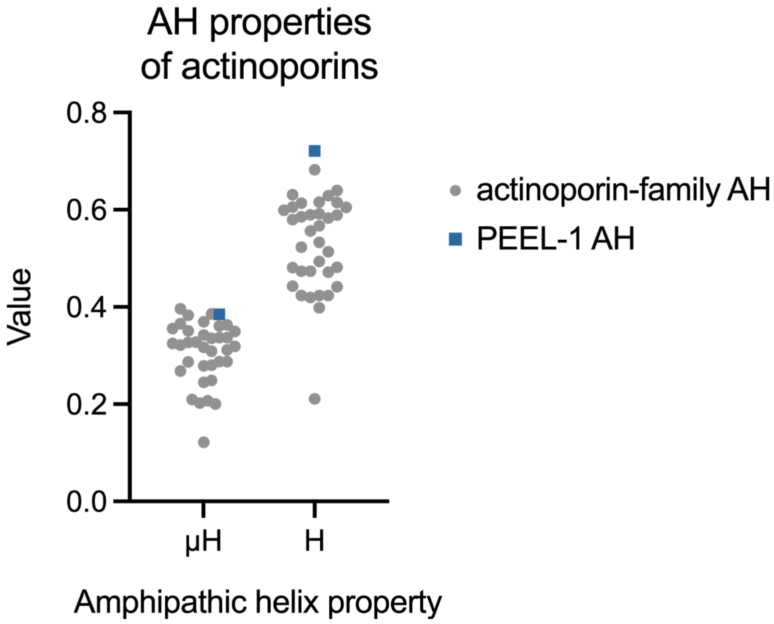

S9 Fig. Amphipathic helix properties of Actinoporin toxins.

Hydrophobic moment (μH) and hydrophobicity (H) of the amphipathic helix of 35 Actinoporin toxin proteins (data acquired from Macrander and Daly, 2016 [29]) (gray circles) and the predicted PEEL-1 amphipathic helix (blue square). Underlying data are available in S2 Data.
