# Supplementary material for: An animal toxin-antidote system kills cells by creating a novel cation channel
Source: PLoS Biol. 2025 May 27;23(5):e3003182. doi: 10.1371/journal.pbio.3003182 (PMC12136403; doi:10.1371/journal.pbio.3003182)
Supplement: S10 Fig — Cytotoxicity of PEEL-1 C-terminal truncations assayed alone (blue bars) or with PMPL-1 (yellow bars) in HEK293T cell transfections. The number of amino acids removed from the C-terminus is indicated (ex. “−28” means 28 amino acids were removed). Data from PEEL-1 WT, −28, −39, and −65 are from Fig 4C. Toxicity is lost upon removal of the −40 residue (Ala135). Statistical tests done using multiple unpaired t-tests with Holm-Šídák test, comparing each PEEL-1 alone to PEEL-1 and PMPL-1 (*p < 0.05; **p < 0.01; ***p < 0.001). Underlying data are available in S2 Data. (PDF) [file pbio.3003182.s010.pdf]

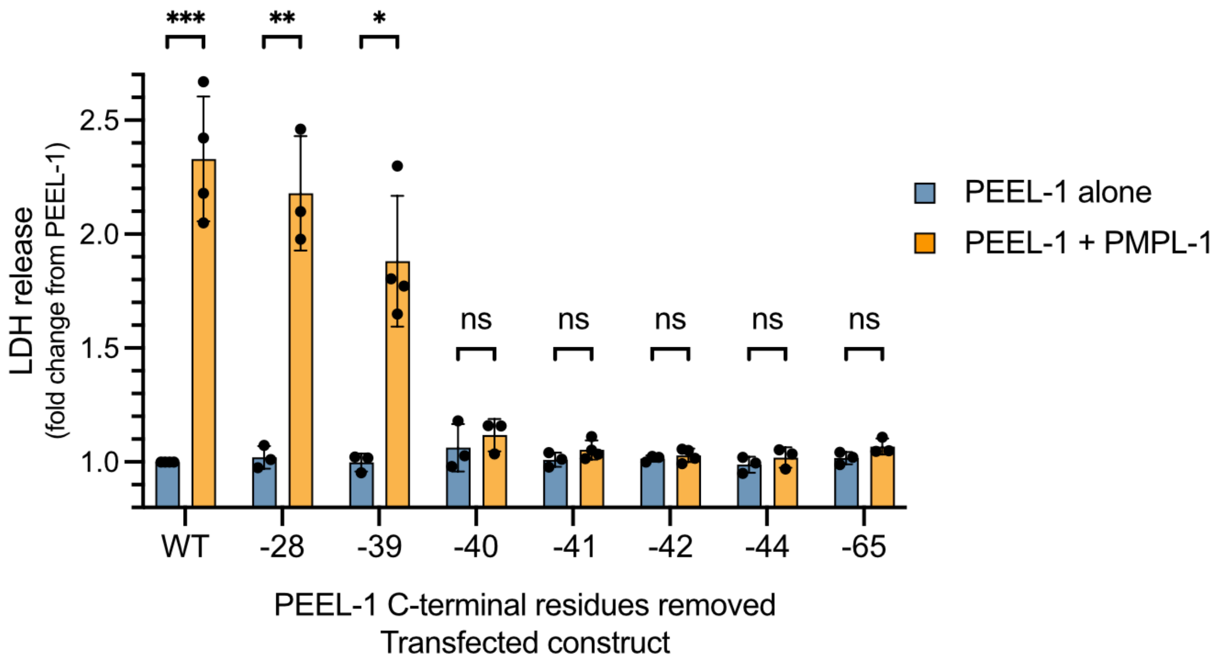

S10 Fig. Toxicity of PEEL-1 C-terminal truncations.

Cytotoxicity of PEEL-1 C-terminal truncations assayed alone (blue bars) or with PMPL-1 (yellow bars) in HEK293T cell transfections. The number of amino acids removed from the C-terminus is indicated (ex. -28 means 28 amino acids were removed). Data from PEEL-1 WT, -28, -39, and -65 are from Fig 4C. Toxicity is lost upon removal of the -40 residue (Ala135). Statistical tests done using multiple unpaired t-tests with Holm-Šídák test, comparing each PEEL-1 alone to PEEL-1 and PMPL-1 (*, $p < 0.05$; **, $p < 0.01$; ***, $p < 0.001$). Underlying data are available in S2 Data.
