# Supplementary material for: An animal toxin-antidote system kills cells by creating a novel cation channel
Source: PLoS Biol. 2025 May 27;23(5):e3003182. doi: 10.1371/journal.pbio.3003182 (PMC12136403; doi:10.1371/journal.pbio.3003182)
Supplement: S11 Fig — (A) Schematic of electrophysiology experiment. High intracellular potassium (140 mM K+/8.6 mM Na+) and high extracellular sodium (145 mM Na+/4 mM K+) solutions are used. Currents elicited by a family of 0.5 s voltage steps from a −30 mV holding potential, from −100 mV to 60 mV, in 10 mV increments. Current traces are normalized to capacitance (pF) and not leak-subtracted. (B) Representative traces from untransfected HEK293 cells (top) and HEK293 cells transfected with peel-1::eGFP (middle) or pmpl-1::mCherry (bottom). Scale bar shown (bottom-right). (C) Representative traces of tetracycline-inducible cells lines. Control cells (left) have stable expression of eGFP and inducible expression of pmpl-1::mCherry. Experimental cells (right) have stable expression of peel-1::eGFP and inducible expression of pmpl-1::mCherry. Recordings of cell lines without induction (top), 6–7 hrs after tetracycline addition (middle), and 28 hrs after tetracycline addition (bottom) are shown. (PDF) [file pbio.3003182.s011.pdf]

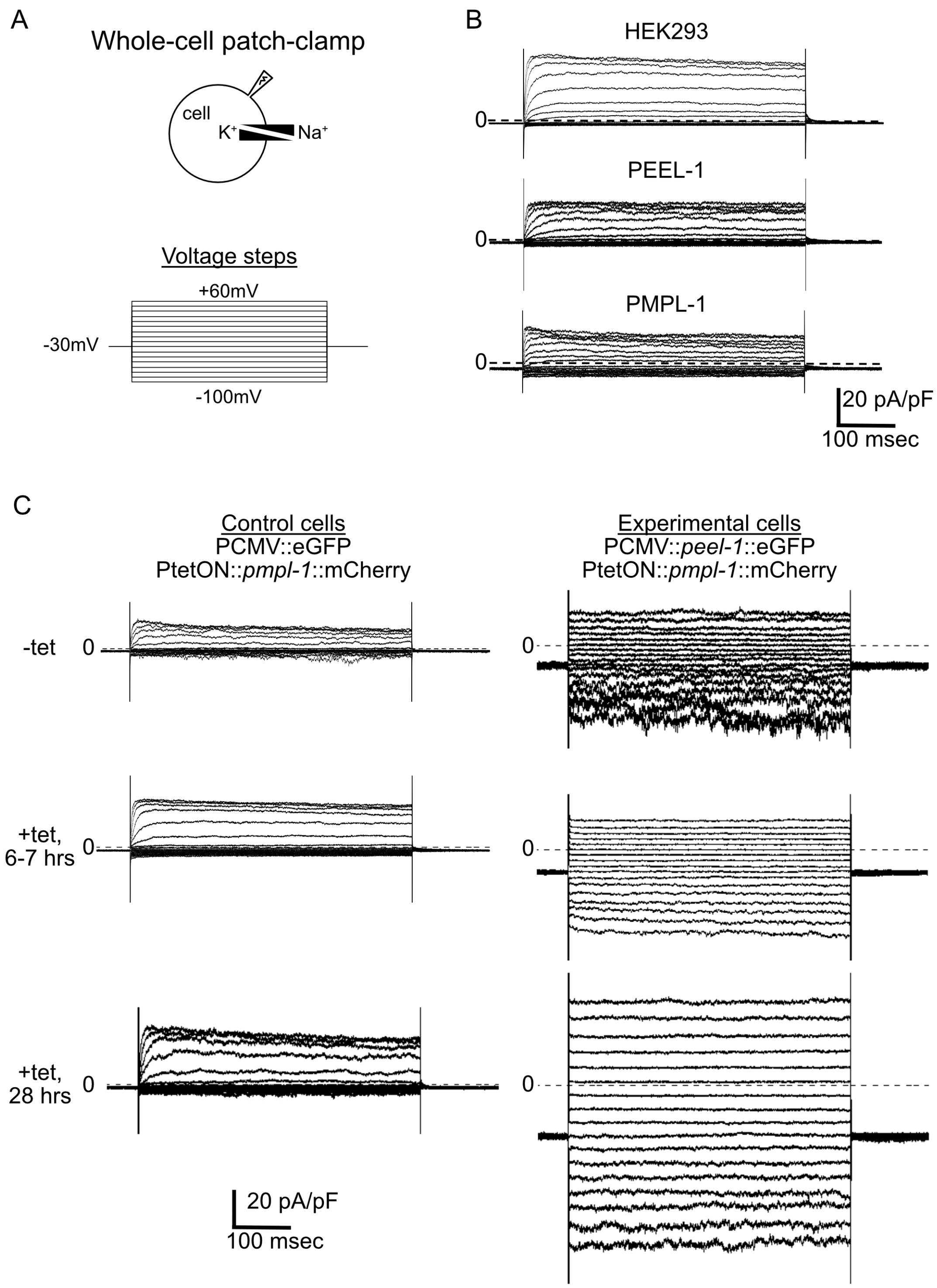

S11 Fig. Raw traces from HEK293 electrophysiology experiments.

(A) Schematic of electrophysiology experiment. High intracellular potassium (140 mM K^+/ 8.6 mM Na^+) and high extracellular sodium (145 mM Na^+/ 4 mM K^+) solutions are used. Currents elicited by a family of 0.5 second voltage steps from a -30 mV holding potential, from -100 mV

to 60 mV, in 10 mV increments. Current traces are normalized to capacitance (pF) and not leak-subtracted. **(B)** Representative traces from untransfected HEK293 cells (top) and HEK293 cells transfected with *peel-1*::eGFP (middle) or *pmpl-1*::mCherry (bottom). Scale bar shown (bottom-right). **(C)** Representative traces of tetracycline-inducible cells lines. Control cells (left) have stable expression of eGFP and inducible expression of *pmpl-1*::mCherry. Experimental cells (right) have stable expression of *peel-1*::eGFP and inducible expression of *pmpl-1*::mCherry. Recordings of cell lines without induction (top), 6-7 hours after tetracycline addition (middle), and 28 hours after tetracycline addition (bottom) are shown.
