# Supplementary material for: An animal toxin-antidote system kills cells by creating a novel cation channel
Source: PLoS Biol. 2025 May 27;23(5):e3003182. doi: 10.1371/journal.pbio.3003182 (PMC12136403; doi:10.1371/journal.pbio.3003182)
Supplement: S12 Fig — (A) Live-cell images of Control cells (CMV-driven eGFP; tetON::pmpl-1::mCherry) and Experimental cells (CMV-driven peel-1::eGFP; tetON::pmpl-1::mCherry). Cell lines are shown without tetracycline (−tet) and 24 hrs after tetracycline addition (+tet (24 hrs)). Insets in −tet conditions show leaky expression of PMPL-1 in both cell lines (LUT adjusted within inset). Images show successful tetracycline-inducible expression of PMPL-1::mCherry and efficient tetracycline-induced killing in experimental cells but not control cells. Arrows and inset in experimental cells +tet (24 hrs) show examples of swollen cells. Exposure time in the green channel is different between cell lines. Scale bar = 40 µm. (B) Time course of toxicity after addition of tetracycline to experimental cells. Noticeable cell swelling is seen after 6 hrs (arrows). Some acute swelling may also be visible at 4 hrs after addition of tetracycline. Scale bar = 20 µm. (PDF) [file pbio.3003182.s012.pdf]

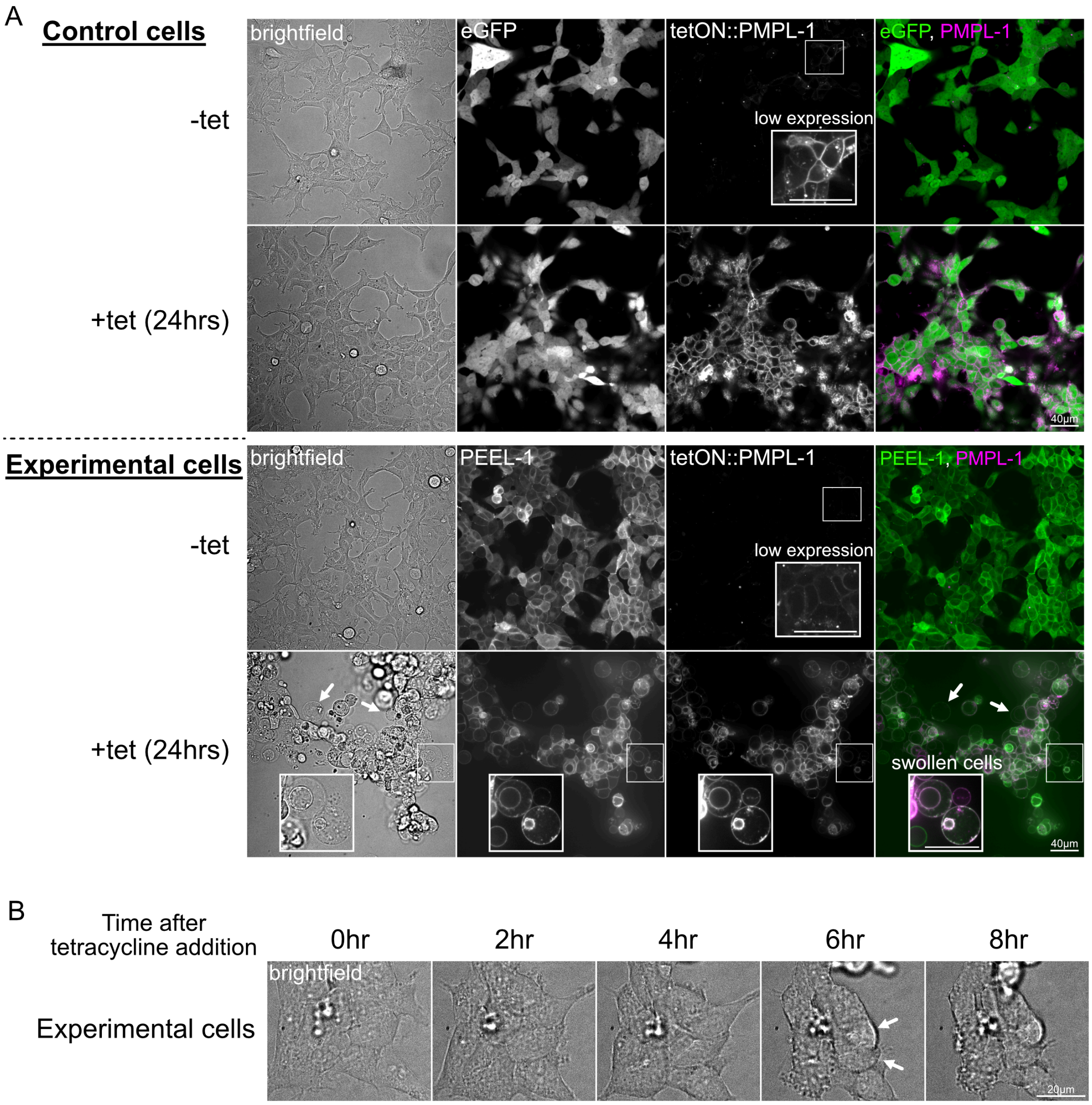

S12 Fig. Tetracycline-induced toxicity in stable cell lines.

(A) Live-cell images of Control cells (CMV-driven eGFP; tetON::*pmpl-1*::mCherry) and Experimental cells (CMV-driven *peel-1*::eGFP; tetON::*pmpl-1*::mCherry). Cell lines are shown without tetracycline (-tet) and 24 hours after tetracycline addition (+tet (24hrs)). Insets in -tet conditions show leaky expression of PMPL-1 in both cell lines (LUT adjusted within inset). Images show successful tetracycline-inducible expression of PMPL-1::mCherry and efficient tetracycline-induced killing in experimental cells but not control cells. Arrows and inset in experimental cells +tet (24 hrs) show examples of swollen cells. Exposure time in the green channel is different between cell lines. Scale bar = 40 µm. **(B)** Time course of toxicity after addition of tetracycline to experimental cells. Noticeable cell swelling is seen after 6 hours (arrows). Some acute swelling may also be visible at 4 hours after addition of tetracycline. Scale bar = 20 µm.
