# Supplementary material for: An animal toxin-antidote system kills cells by creating a novel cation channel
Source: PLoS Biol. 2025 May 27;23(5):e3003182. doi: 10.1371/journal.pbio.3003182 (PMC12136403; doi:10.1371/journal.pbio.3003182)
Supplement: S13 Fig — Current–voltage plots of Experimental cells without tetracycline in different ionic conditions to test permeabilities of the indicated ions. Permeable ions have greater inward currents (Na+, K+, Cs+; closed symbols) compared to impermeable ions (NMDG+, Ca2+, Cl−; open symbols) at negative voltages. Mean with SEM is shown. These data are summarized in Fig 5E. Underlying data are available in S2 Data. (PDF) [file pbio.3003182.s013.pdf]

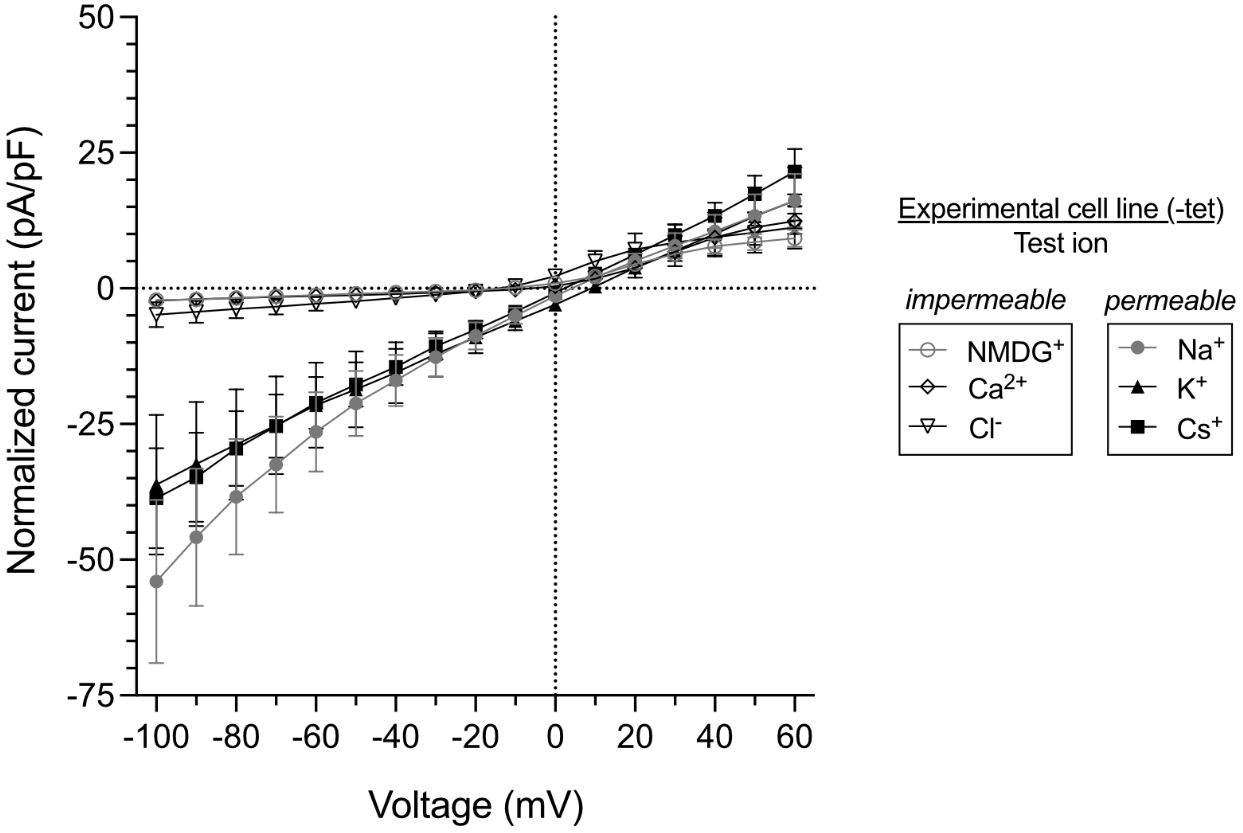

S13 Fig. Uninduced Experimental cells are permeable to monovalent cations.

Current-voltage plots of Experimental cells without tetracycline in different ionic conditions to test permeabilities of the indicated ions. Permeable ions have greater inward currents (Na^+, K^+, Cs^+; closed symbols) compared to impermeable ions ($NMDG^+$, Ca^{2+}, Cl^-; open symbols) at negative voltages. Mean with SEM is shown. These data are summarized in Fig 5E. Underlying data are available in S2 Data.
