# Supplementary material for: An animal toxin-antidote system kills cells by creating a novel cation channel
Source: PLoS Biol. 2025 May 27;23(5):e3003182. doi: 10.1371/journal.pbio.3003182 (PMC12136403; doi:10.1371/journal.pbio.3003182)
Supplement: S14 Fig — AlphaFold2 prediction of the PEEL-1 pentameric structure is shown. Two angles are shown: (A–B) top view, showing the predicted extracellular face of the complex, and (C–D) side view, in the plane of the lipid bilayer. (A and C) Ribbon diagram with the amphipathic helix colored in blue creating the lining of a pore-like region. (B) Surface representation of electrostatic predictions (red = negative charge, blue = positive charge). An uninterrupted hole can be seen through the structure, with a ring of negative charge from five D109 residues. (D) Surface representation of hydrophobicity (yellow = hydrophobic, cyan = hydrophilic). PDB file available in S3 Data. (E) Cytotoxicity of mutants which eliminate the predicted ring of negative charge at the top of the complex via a D109A mutation. Plot shows mean with SD. Statistics performed using multiple unpaired t-tests with Holm-Šídák test. All tested comparisons are shown. Underlying data for (E) are available in S2 Data. (PDF) [file pbio.3003182.s014.pdf]

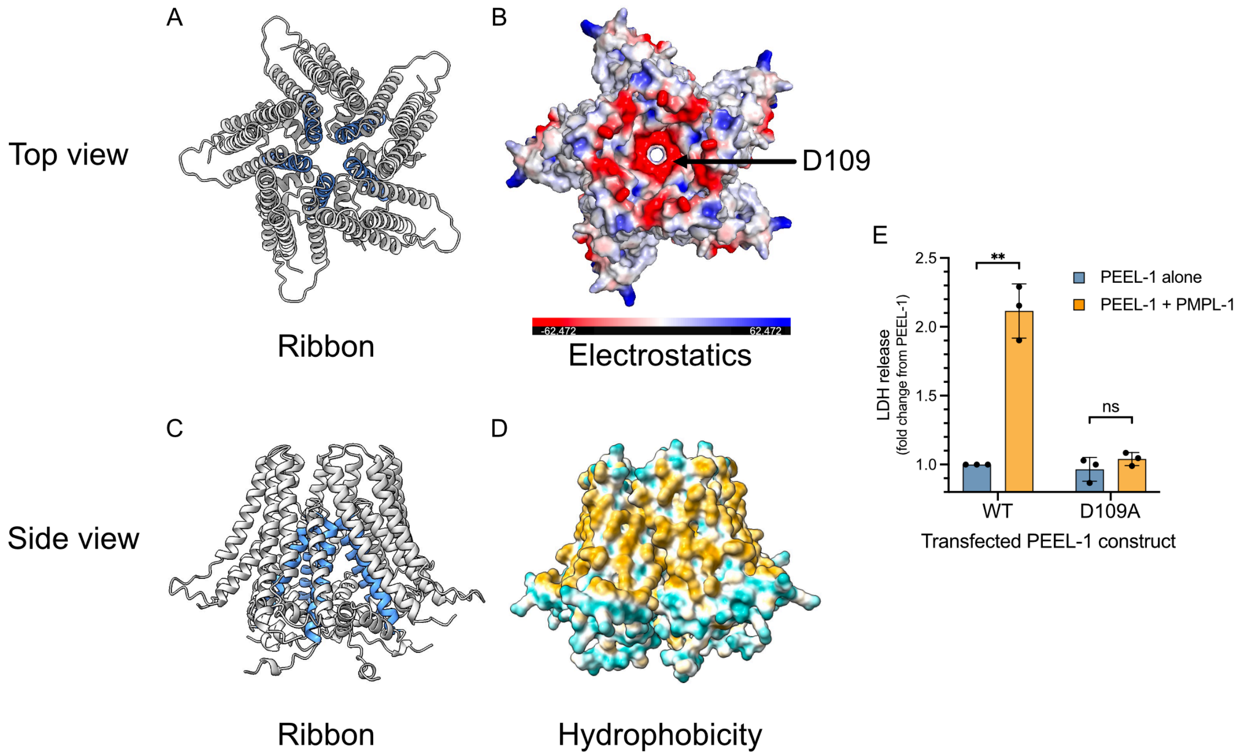

S14 Fig. Predicted PEEL-1 pentameric structure.

AlphaFold2 prediction of the PEEL-1 pentameric structure is shown. Two angles are shown: **(A-B)** top view, showing the predicted extracellular face of the complex, and **(C-D)** side view, in the plane of the lipid bilayer. **(A and C)** Ribbon diagram with the amphipathic helix colored in blue creating the lining of a pore-like region. **(B)** Surface representation of electrostatic predictions (red = negative charge, blue = positive charge). An uninterrupted hole can be seen through the structure, with a ring of negative charge from five D109 residues. **(D)** Surface representation of hydrophobicity (yellow = hydrophobic, cyan = hydrophilic). PDB file available in S3 Data. **(E)** Cytotoxicity of mutants which eliminate the predicted ring of negative charge at the top of the complex via a D109A mutation. Plot shows mean with SD. Statistics performed using multiple unpaired t-tests with Holm-Šídák test. All tested comparisons are shown. Underlying data for (E) are available in S2 Data.
