# Supplementary material for: An animal toxin-antidote system kills cells by creating a novel cation channel
Source: PLoS Biol. 2025 May 27;23(5):e3003182. doi: 10.1371/journal.pbio.3003182 (PMC12136403; doi:10.1371/journal.pbio.3003182)
Supplement: S15 Fig — (A) SDS–PAGE gels of indicated constructs after purification (P; Coomassie Blue stain and anti-MBP western blot) and after incorporation into liposomes (L; anti-MBP western blots). Liposomes were collected after flotation through a density gradient before use in synthetic bilayer experiments. The top fraction contains liposomes (L) and the bottom fraction (B) contains unincorporated protein. Monomers (m), oligomers or aggregates (o), and truncation products of PEEL-1 (t) are indicated. The bottom bands seen in both MBP-tagged PEEL-1 and PMPL-1 purifications are soluble proteins (s) that did not get incorporated into liposomes, likely endogenous MBP (43 kDa) or truncated MBP-tagged protein. (B) PEEL-1 is toxic to bacteria and toxicity requires the amphipathic helix. E. coli C41(DE3) cells with constructs encoding IPTG-inducible expression of either MBP::PEEL-1 (left) or MBP::PEEL-1(−65aa) (right). Images of cultures are taken after shaking at 18 °C overnight, with or without 0.5 mM IPTG. Cultures appear lysed when full-length PEEL-1 is expressed but not PEEL-1(−65aa) which lacks the PEEL-1 AH. Western blot (anti-MBP) of corresponding whole-cell lysates are shown (bottom), confirming higher expression of MBP::PEEL-1(−65aa). This experiment was repeated four times and yielded similar results. Original uncropped blots available in S1 Raw images. (PDF) [file pbio.3003182.s015.pdf]

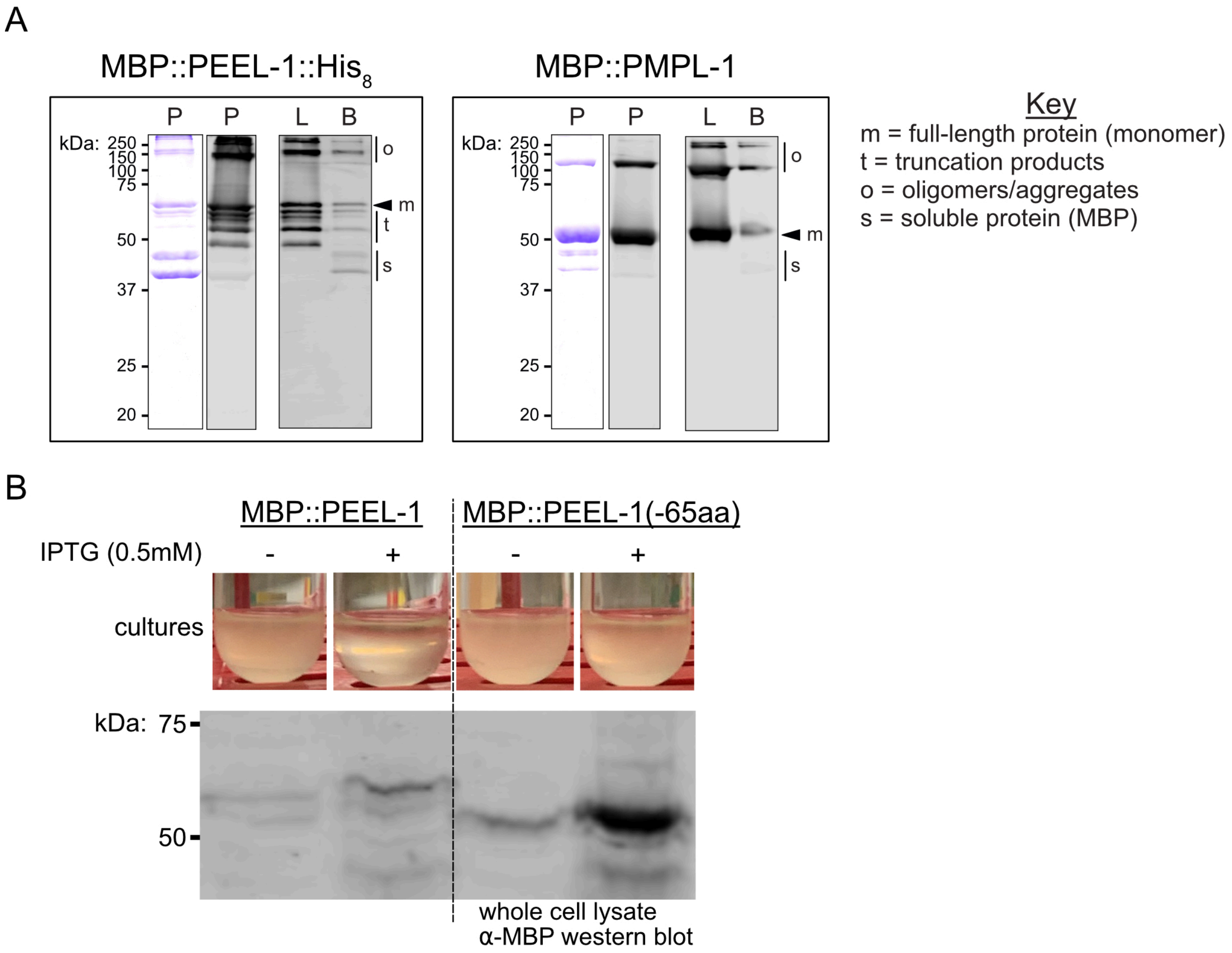

S15 Fig. PEEL-1 and PMPL-1 purification and liposomes.

(A) SDS-PAGE gels of indicated constructs after purification (P; Coomassie Blue stain and anti-MBP western blot) and after incorporation into liposomes (L; anti-MBP western blots). Liposomes were collected after flotation through a density gradient before use in synthetic bilayer experiments. The top fraction contains liposomes (L) and the bottom fraction (B) contains unincorporated protein. Monomers (m), oligomers or aggregates (o), and truncation products of PEEL-1 (t) are indicated. The bottom bands seen in both MBP-tagged PEEL-1 and PMPL-1 purifications are soluble proteins (s) that did not get incorporated into liposomes, likely endogenous MBP (43 kDa) or truncated MBP-tagged protein. **(B)** PEEL-1 is toxic to bacteria and toxicity requires the amphipathic helix. *E. coli* C41(DE3) cells with constructs encoding IPTG-inducible expression of either MBP::PEEL-1 (left) or MBP::PEEL-1(-65aa) (right). Images of cultures are taken after shaking at 18°C overnight, with or without 0.5 mM IPTG. Cultures appear lysed when full-length PEEL-1 is expressed but not PEEL-1(-65aa) which lacks the PEEL-1 AH. Western blot (anti-MBP) of corresponding whole-cell lysates are shown (bottom), confirming higher expression of MBP::PEEL-1(-65aa). This experiment was repeated four times and yielded similar results. Original uncropped blots available in S1 Raw Images.
