# Supplementary material for: An animal toxin-antidote system kills cells by creating a novel cation channel
Source: PLoS Biol. 2025 May 27;23(5):e3003182. doi: 10.1371/journal.pbio.3003182 (PMC12136403; doi:10.1371/journal.pbio.3003182)
Supplement: S16 Fig — Conductance traces through artificial planar lipid bilayers are shown. (A) Bilayer alone without addition of liposomes, (B) bilayer with PEEL-1 liposomes added (transient spikes indicate successful liposome fusions), (C) bilayer with PMPL-1 liposomes added, and (D–E) two independent experiments of bilayers with PEEL-1 and PMPL-1 liposomes added. An all-point histogram is shown for each trace (right, 2 pS bin width, normalized based on probability density). Channel activity after addition of PEEL-1 and PMPL-1 was observed in 8 independent experiments. A voltage of −180 mV was applied to the bilayer for liposome fusions in all experiments. After observing channel activity, a voltage of +180 mV was applied since channel activity was more stable at positive voltages. Bottom two panels show traces at +180 mV. Scale bar = 5 s. Underlying data are available in S2 Data. (PDF) [file pbio.3003182.s016.pdf]

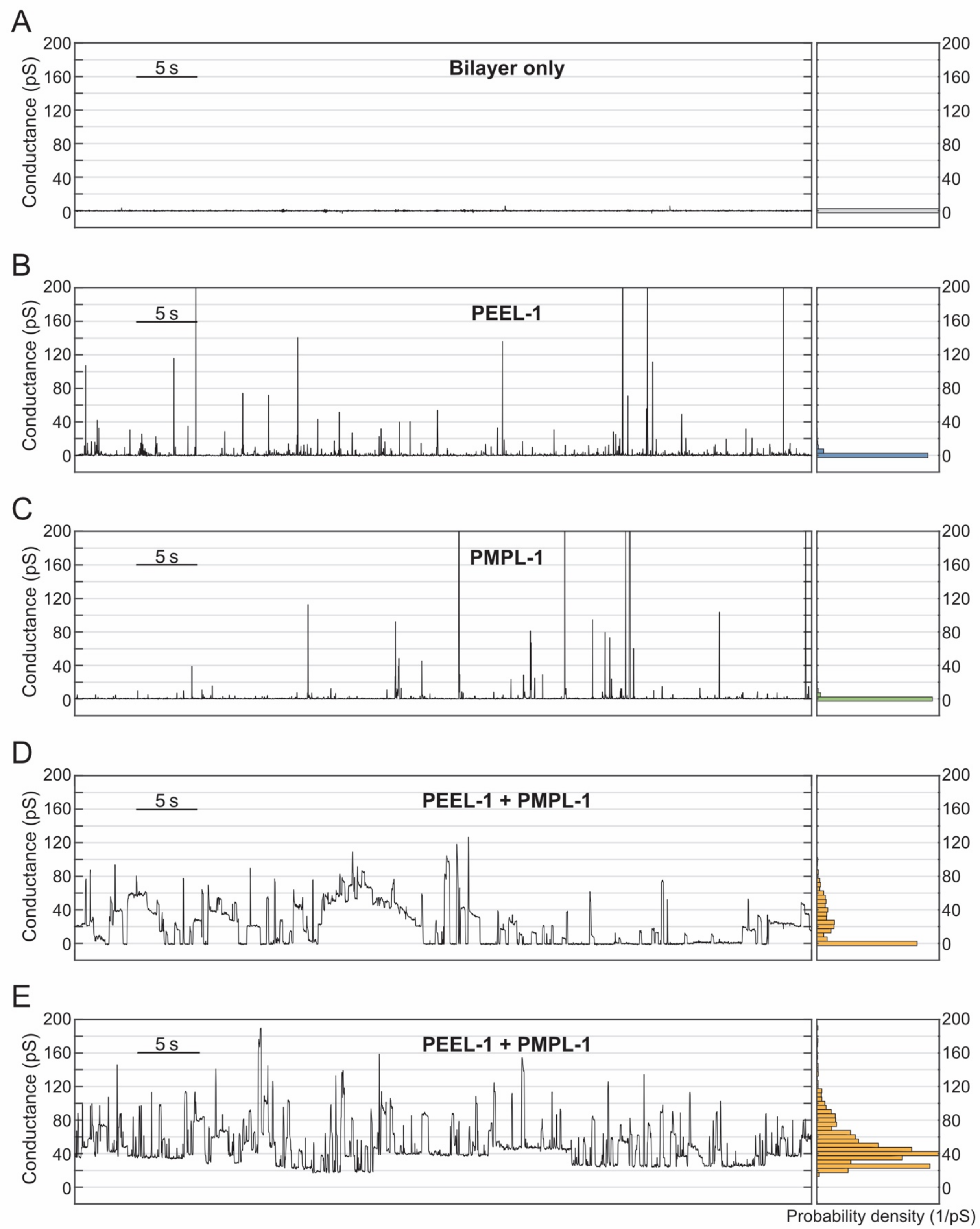

S16 Fig. Purified PEEL-1 and PMPL-1 conduct ions through planar lipid bilayers.

Conductance traces through artificial planar lipid bilayers are shown. **(A)** Bilayer alone without addition of liposomes, **(B)** bilayer with PEEL-1 liposomes added (transient spikes indicate

successful liposome fusions), **(C)** bilayer with PMPL-1 liposomes added, and **(D-E)** two independent experiments of bilayers with PEEL-1 and PMPL-1 liposomes added. An all-point histogram is shown for each trace (right, 2 pS bin width, normalized based on probability density). Channel activity after addition of PEEL-1 and PMPL-1 was observed in 8 independent experiments. A voltage of -180 mV was applied to the bilayer for liposome fusions in all experiments. After observing channel activity, a voltage of +180 mV was applied since channel activity was more stable at positive voltages. Bottom two panels show traces at +180 mV. Scale bar = 5 seconds. Underlying data are available in S2 Data.
