# Supplementary material for: An animal toxin-antidote system kills cells by creating a novel cation channel
Source: PLoS Biol. 2025 May 27;23(5):e3003182. doi: 10.1371/journal.pbio.3003182 (PMC12136403; doi:10.1371/journal.pbio.3003182)
Supplement: S1 Table — (PDF) [file pbio.3003182.s017.pdf]

S1 Table. *C. elegans* strains used in this study.

Strain ID	Strain genotype	Source
N2	N2	CGC
XZ1372	yakTi4[hsp-16.41p::eGFP::his-44 , NeoR] I ; oxSi507[hsp-16.41p::peel-1, Cb-unc-119] II ; oxSi280[hsp-16.41p::peel-1, Cb-unc-119] IV	[1]
XZ1047	oxSi507[hsp-16.41p::peel-1, Cb-unc-119] II ; unc-119(ed9) III ; oxSi280[hsp-16.41p::peel-1, Cb-unc-119] IV ; him-5(e1490) V	[1]
XZ103	oxSi507[hsp-16.41p::peel-1, Cb-unc-119] II ; oxSi280[hsp-16.41p::peel-1, Cb-unc-119] IV ; pmpl-1(yak103) X	this study
XZ2283	oxSi507[hsp-16.41p::peel-1, Cb-unc-119] II ; oxSi280[hsp-16.41p::peel-1, Cb-unc-119] IV ; F47B7.1(yak52) X	this study
AFS216	zeel-1(tm3419) I peel-1(cle6) I	Aaron Severson
XZ1177	oxSi507[hsp-16.41p::peel-1, Cb-unc-119] II ; unc-119(ed9) III ; oxSi280[hsp-16.41p::peel-1, Cb-unc-119] IV ; him-5(e1490) V ; pmpl-1(yak52) X	this study
XZ1307	oxSi507[hsp-16.41p::peel-1, Cb-unc-119] II ; oxSi280[hsp-16.41p::peel-1, Cb-unc-119] IV ; him-5(e1490) V ; pmpl-1(yak103) X	this study
EG1000	dpy-5(e61) I ; rol-6(e187) II ; lon-1(e1820) III	Erik M. Jorgensen
EG1020	bli-6(sc16) IV ; dpy-11(e224) V ; lon-2(e678) X	Erik M. Jorgensen
EG8040	oxTi302[Peft-3::mCherry cb-unc-119(+)] I ; oxTi75[Peft-3::GFP::H2B::tbb-2utr unc-18(+)] II ; oxTi411[Peft-3::TdTomato::H2B::unc-54 cb-unc-119(+)] III ; him-8(e1489) IV	Jorgensen lab
EG8041	oxTi76[Peft-3::GFP::H2B::tbb-2utr unc-18(+)] IV ; oxTi405[Peft-3::TdTomato::H2B::unc-54 cb-unc-119(+)] V him-5(e1490) V ; oxTi421[Peft-3::mCherry cb-unc-119(+)] X	Jorgensen lab
XZ2194	pmpl-1 (yak103) X	this study
XZ2103	oxSi507[hsp-16.41p::peel-1, Cb-unc-119] II ; ced-3(n717) IV ; him-5(e1490) V	this study
XZ2102	oxSi507[hsp-16.41p::peel-1, Cb-unc-119] II ; ced-5(n1812) IV	this study
XZ2096	oxSi507[hsp-16.41p::peel-1, Cb-unc-119] II ced-2(n1994) IV	this study
XZ2254	yakEx195[pmpl-1p::GFP; myo-2p::mcherry; myo-3p::mcherry; rab-3p::mCherry]	this study
XZ2276	pmpl-1(yak103) X ; yakEx203[exp-3p::peel-1::GFP, myo-3p::mCherry]	this study
XZ2633	pmpl-1(yak103) X ; yakEx275[exp-3p::peel-1::GFP, exp-3p::pmpl-1::GFP, myo3p::mCherry]	this study
XZ2551	yakEx264[hsp-16.41p::peel-1(-28aa), cc::GFP]	this study
XZ2634	yakEx276[hsp-16.41p::peel-1(-39aa), cc::GFP]	this study
XZ2548	yakEx263[hsp-16.41p::peel-1(-65aa), cc::GFP]	this study
XZ2454	pmpl-1(yak103) X ; hjSi56[Pvha-6::3xFLAG::TEV::GFP::dgat-2::let-858 3' UTR] IV ; yakEx243[vha-6p::peel-1::tagRFP, myo-2p::mCherry]	this study
XZ2452	hjSi56[Pvha-6::3xFLAG::TEV::GFP::dgat-2::let-858 3' UTR] IV ; yakEx242[vha-6p::pmpl-1::tagRFP, myo-2p::mCherry]	this study

Reference

1. Crawford MW, Posch G, Cattin-Ortolá J, Topalidou I, Ailion M. Mutations in the NXF-1:NXT-1 mRNA export complex affect gene-expression driven by the hsp-16.41 promoter. MicroPublication Biol. 2023. doi:10.17912/micropub.biology.000918
