# Supplementary material for: An animal toxin-antidote system kills cells by creating a novel cation channel
Source: PLoS Biol. 2025 May 27;23(5):e3003182. doi: 10.1371/journal.pbio.3003182 (PMC12136403; doi:10.1371/journal.pbio.3003182)
Supplement: S2 Table — (PDF) [file pbio.3003182.s018.pdf]

S2 Table. Constructs used in this study.

**Construct ID**	**Description**	**resistance**	**sequenced?**	**notes**		for expression in…
pLC4	exp-3p::pmpl-1::tagRFP::tbb-2 3'UTR	carb				worm
pLC6	exp-3p::peel-1::GFP::tbb-2 3'UTR	carb				worm
pLC26	MBP::TEV::peel-1	carb	sequenced			bacteria
pLC28	MBP::TEV::pmpl-1	carb	sequenced			bacteria
pLC31	exp-3p::pmpl-1::GFP tbb-2 3'UTR	carb				worm
pLC37	PMP3(S. cerevisiae)::mCherry_N1	kan	sequenced			mammalian
pLC38	mCherry::zeel-1_N1	kan				mammalian
pLC54	pmpl-1::eGFP_N1	kan				mammalian
pLC65	MBP::TEV::peel-1	carb+chlor	sequenced			bacteria
pLC67	MBP::TEV::pmpl-1	carb+chlor	sequenced			bacteria
pLC79	tetON::pmpl-1::mCherry_pFTSH	carb	sequenced			mammalian
pLC84	exp-3p::pmpl-1::tagRFP::tbb-2 3'UTR	carb				worm
pLC103	vha-6p::pmpl-1::tagRFP::tb-2 3'UTR	carb				worm
pLC113	vha-6p::peel-1::tagRFP::tbb-2 3'UTR	carb				worm
pLC122	pmpl-2::mCherry_N1	kan	sequenced			mammalian
pLC123	pmpl-1(A47T)::mCherry_N1	kan	sequenced			mammalian
pLC124	peel-1(S124F)::eGFP_N1	kan	sequenced			mammalian
pLC172	hsp-16.41p::peel-1(-28aa)::let-858 3'UTR	carb	sequenced			worm
pLC173	hsp-16.41p::peel-1(-65aa)::let-858 3'UTR	carb	sequenced			worm
pLC174	peel-1(-28aa)_N1	kan	sequenced			mammalian
pLC175	peel-1(-65aa)_N1	kan	sequenced			mammalian
pLC226	peel-1(-39aa)_N1	kan	sequenced			mammalian
pLC227	MBP::TEV::peel-1(-65aa)	carb	sequenced			bacteria
pLC294	MBP::TEV::peel-1 (-65aa)	carb/chlor	sequenced			bacteria
pLC297	peel-1(-42aa)_N1	kan	sequenced			mammalian
pLC298	peel-1(-44aa)_N1	kan	sequenced			mammalian
pLC304	peel-1(-41aa)_N1	kan	sequenced			mammalian
pLC305	peel-1(-40aa)_N1	kan	sequenced			mammalian
pLC345	peel-1::eGFP::ER-ret(GBR1 C-tail)_N1	kan	sequenced			mammalian
pLC363	peel-1(S124V)::eGFP_N1	kan	sequenced			mammalian
pLC365	pmpl-1::mCherry::ER-ret(GBR1 C-tail)_N1	kan	sequenced			mammalian
pLC370	MBP::TEV::peel-1::8X His	carb	sequenced			bacteria
pLC376	MBP::TEV::peel-1::8X His	carb/chlor	sequenced			bacteria
pLC385	peel-1(D109A)::eGFP_N1	kan	sequenced			mammalian
pLC395	peel-1(L115Q)::eGFP_N1	kan	sequenced			mammalian
pLC396	peel-1(L118Q)::eGFP_N1	kan	sequenced			mammalian
pLC397	peel-1(L122Q)::eGFP_N1	kan	sequenced			mammalian
pLC398	peel-1(L126Q)::eGFP_N1	kan	sequenced			mammalian
pLC438	peel-1(L118Q,S124V)::eGFP_N1	kan	sequenced			mammalian
pLC439	peel-1(L118Q,L126Q)::eGFP_N1	kan	sequenced			mammalian
pLC440	peel-1(S124V,L126Q)::eGFP_N1	kan	sequenced			mammalian
pLC475	hsp-16.41p::peel-1(-39aa)::let-858 3'UTR	carb	sequenced			mammalian
mCherry-KDEL	mCherry-KDEL	kan		gift from Suzanne Hoppins		mammalian
pGP9	peel-1::eGFP_N1	kan	sequenced			mammalian
pGP10	pmpl-1::mCherry_N1	kan	sequenced			mammalian
pPD97/98	cc::GFP (unc-122p::GFP)	carb		gift from Piali Sengupta		worm
pCFJ90	Pmyo-2::mCherry::unc-54 3'UTR	carb				worm
pCFJ104	Pmyo-3::mCherry::unc-54 3'UTR	carb				worm
pGH8	Prab-3::mCherry::unc-54 3'UTR	carb				worm
pBS_SK	pBluescript	carb				worm
pCFJ150	destination vector (4-1-2-3)	carb				
eGFP_N1	eGFP_N1	kan		gift from Suzanne Hoppins		mammalian
mCherry_N1	mCherry_N1	kan		gift from Suzanne Hoppins		mammalian
pOG44	FLP recombinase	carb		gift from Nancy Maizels		mammalian
pFTSH	vector backbone, tetON expression	carb		gift from Nancy Maizels		mammalian
