# Supplementary material for: An animal toxin-antidote system kills cells by creating a novel cation channel
Source: PLoS Biol. 2025 May 27;23(5):e3003182. doi: 10.1371/journal.pbio.3003182 (PMC12136403; doi:10.1371/journal.pbio.3003182)

Original blots used for Figure 5D

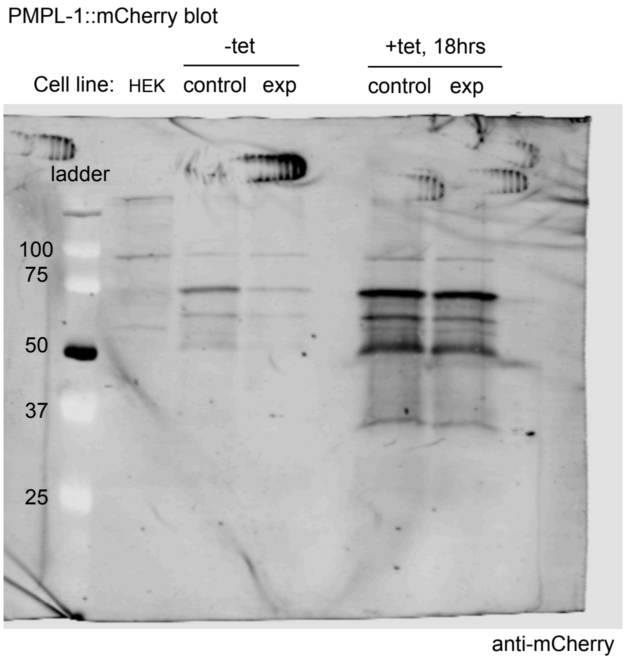

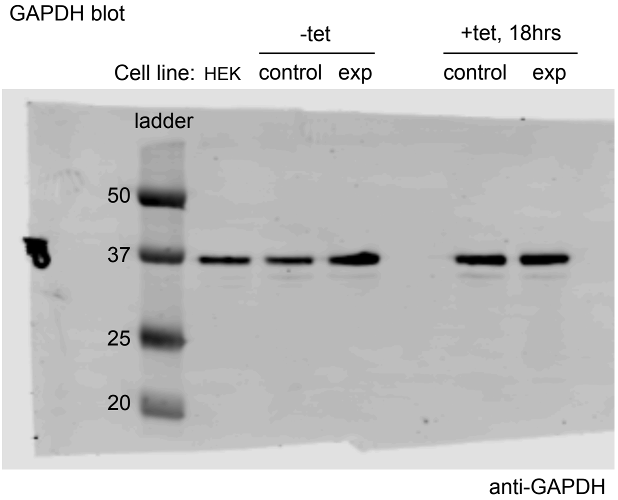

Original blot used for Figure S15A

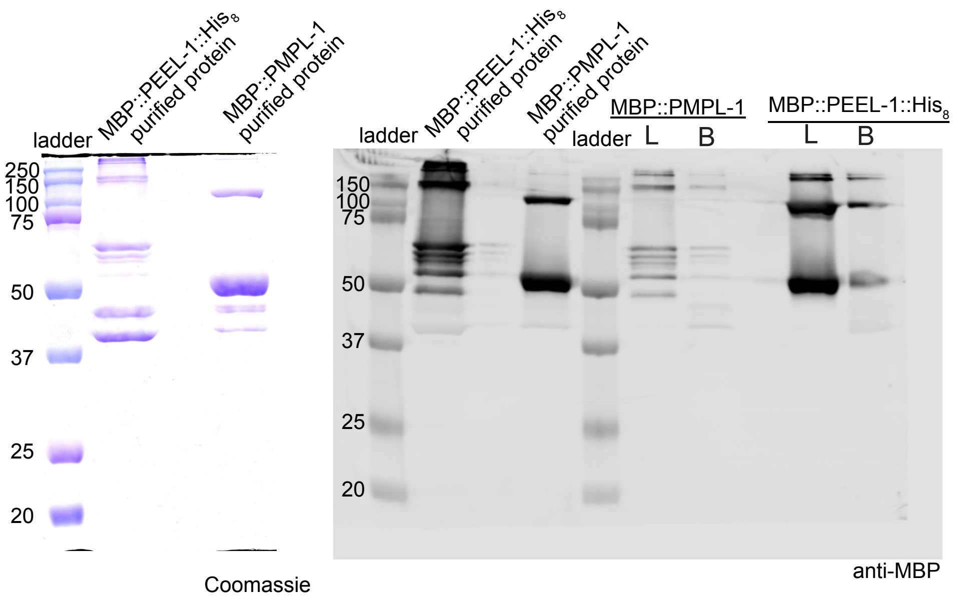

Original blot used for Figure S15B

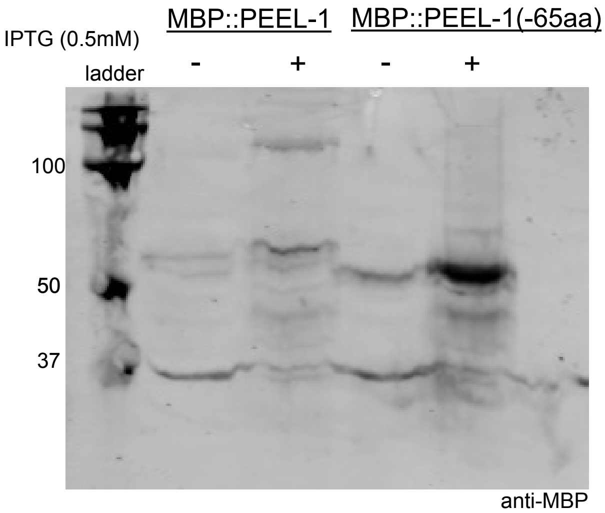

Supplement: S1 Raw images — (PDF) [file pbio.3003182.s023.pdf]
