# Supplementary material for: An animal toxin-antidote system kills cells by creating a novel cation channel
Source: PLoS Biol. 2025 May 27;23(5):e3003182. doi: 10.1371/journal.pbio.3003182 (PMC12136403; doi:10.1371/journal.pbio.3003182)
Supplement: Attachment — Submitted filename: 149831_1_rebuttal_3202437_smnm69.pdf [file pbio.3003182.s024.pdf]

Full Revision

Review COMMONS

Manuscript number: RC-2024-02646
Corresponding author(s): Michael Ailion

[Please use this template only if the submitted manuscript should be considered by the affiliate journal as a full revision in response to the points raised by the reviewers.

If you wish to submit a preliminary revision with a revision plan, please use our "Revision Plan" template. ***It is important to use the appropriate template to clearly inform the editors of your intentions.****]*

1. General Statements [optional]

This section is optional. Insert here any general statements you wish to make about the goal of the study or about the reviews.

The goal of this study was to determine the molecular and cellular mechanism of toxicity of a selfish genetic element: *peel-1 zeel-1* from *C. elegans*. While *peel-1 zeel-1* has been the best understood toxin-antidote (TA) system in animals for over a decade, the mechanism of toxin and antidote activity remained unknown. Here we show that that the PEEL-1 toxin co-opts PMPL-1, a small membrane protein of unknown function, for toxicity. Together, these proteins induce cell death in human cells. Combining electrophysiology and biochemistry, we show that PEEL-1 and PMPL-1 kill cells by creating a novel ion channel. Structural predictions of PEEL-1 oligomers provide a view into how ion channels can be constructed by evolutionarily novel proteins. Our work is the first to define a major part of the molecular mechanism in any animal TA system.

We received three reviews of our manuscript. Reviewers #1 & 3 were very positive and had only minor suggestions that involved changes to the text, but no additional experiments. Reviewer #2 had some suggestions for additional experiments. Though Reviewer #2 stated that “the data demonstrate the presence of a channel,” they felt that “the study lacks deeper characterization of the channel's dynamics and ion-specific current responses.” Reviewer #2 also asked for additional experiments to corroborate proposed structural hypotheses and to more specifically determine how PEEL-1 and PMPL-1 interact. In referee cross-comments, both Reviewers #1 & 3 agreed that the experiments suggested by Reviewer #2 would be interesting, but argued that they are beyond the scope of the current paper. We agree with Reviewers #1 & 3. Importantly, though these additional experiments would provide further mechanistic details about how PEEL-1 and PMPL-1 act, they are not necessary for any of the major conclusions of our paper. Thus, we also think they are outside the scope of the current study. The additional experiments suggested by Reviewer #2 would be technically challenging and very laborious, probably requiring several years to complete. We feel that these experiments are more suited for future work on this project.

2. Point-by-point description of the revisions

This section is mandatory. Please insert a point-by-point reply describing the revisions that were <u>already carried out and included</u> in the transferred manuscript.

We have made changes to the text of the manuscript, but have not changed any of the figures. Specifically, we made edits to the Results section on localization of PEEL-1 and PMPL-1 to clarify that PEEL-1 and PMPL-1 are colocalized at both the ER and plasma membrane and to note that they are also localized to the nuclear envelope-associated ER. We also completely rewrote and expanded the Discussion section, as suggested by the reviewers. We added discussion of possible mechanisms of how PEEL-1 and PMPL-1 may cause cell death, how PMPL-1 may interact directly or indirectly with PEEL-1, how ZEEL-1 may act as an antidote, and how the planar lipid bilayer *in vitro* system could be used to determine more details of the ion channel structure and biophysical properties.

A detailed point-by-point response to the reviewers' comments is appended.

Reviewer #1 (Evidence, reproducibility and clarity (Required)):

This is an interesting paper in which the authors dissect the mechanism by which the peel-1 sperm-based toxin in the peel-1/zeel-1 system works. The toxin is loaded into sperm and kills progeny who fail to inherit it, with zeel1 providing embryonic rescue to carriers. The data are clear. There are points in the paper in which the authors are forced to go a bit out on the edge in terms of hypothesizing how peel-1 works based on low quality structure predictions. That said, they are clear at each of these points that their models are tentative and require further testing. Also, the ionic current and liposome data provide support for their proposed mechanism.

There has been no characterization of Peel1 since its initial characterization in 2011. Partly this is probably due to the fact (from this paper) that while expression of peel1 in C. elegans using a heat shock promoter results in death, expression on its own in mammalian cells has no effects. One possible explanation for this is that other co-factors are needed.

The authors first discover that this is the case. They carry out a genetic screen looking for suppressors of Peel1 toxicity and identify one gene, pmpl1, loss of which blocks peel1-dependent death. Most importantly, co-expression of peel1 and pmpl1 causes death of mammalian 293 cells, which is suppressed by expression of zeel1, providing evidence of specificity. These results are clear and convincing and provide an opportunity to test mechanistic models in which the two proteins are sufficient, on their own, to induce cell death.

These results also help to make sense of some observations in the worm, such as why peel1 does not kill sperm. The authors observe that pmpl1 is expressed at lower levels in developing sperm than it is in other tissues, providing a possible mechanism of protection. A prediction of this hypothesis (not tested in the current paper) is that ectopic expression of pmpl1 in sperm

should (assuming no other unknown suppressors exist) cause their death.

Much of the rest of the manuscript focuses on trying to understand mechanism through localization, mutagenesis informed by predicted structures, and tests of ion conductance changes in cells and artificial lipid membranes.

The authors use the ER retention signal KDEL to explore if Peel1/Pmpl1 act at the plasma membrane. KDEL tagging suppresses toxicity, suggesting it does. The rapid onset of cell pathology, the nature of the pathology (cell swelling), and lack of involvement of the cell death gene ced-3, is also consistent with a novel mechanism.

In Fig. 3 they visualize some of the cell pathology. In a supplement it appears the pmpl1 localizes very strongly along the nuclear envelope-associated ER. Is this worth commenting on a bit more?

We edited the manuscript to point out this observation. Both PEEL-1 and PMPL-1 localize to the nuclear envelope-associated ER (Fig. 2C-D). This localization is more obvious in swelling cells because the ER is fragmented while the nucleus stays intact (Fig. S7). Our data suggest that ER localization is not relevant to toxicity, so to keep the focus on the toxin mechanism we did not discuss nuclear envelope-associated ER further.

The text now reads: "PEEL-1 and PMPL-1 localize to several membrane-bound compartments in HEK293T cells and *C. elegans*. Both proteins localize to the endoplasmic reticulum (ER), nuclear envelope-associated ER, and plasma membrane (PM) (Fig. 2C-D and Fig. S6B-C)."

Electrophysiology suggests that Peel1 might be forming a non-specific monovalent cation channel. At one point they speculate the pathology could be related to unrestricted Na flow into the cell and an osmotic imbalance. But, wouldnt K be flowing out at the same time, counterbalancing any osmotic effect? If Na flux is the key, would it make sense to ask if low extracellular Na, buffered with some other impermeable cation, blocks pathology? This is just a question, not a direct request for an experiment.

Yes, the reviewer is correct that Na+ and K+ both appear to flow through the ion channel, and K+ would be expected to be flowing out of the cell at the same time that Na+ is flowing in. However, at negative membrane potentials, the influx of Na+ is higher than the efflux of K+, so the net current will be inward. Since cells have a negative resting membrane potential under physiological conditions, the Na+ influx will be greater than the K+ efflux in our experiments. We hypothesize that the persistent influx of Na+ will lead to an increase in cytosolic osmolarity, causing influx of water to the cell, and cell swelling. We speculate that there is a failure in the homeostatic mechanisms that normally counteract cell swelling, ultimately leading to cell lysis. One possibility is that the persistent Na+ influx may overwork the ATP-dependent Na+/K+ pump, leading to ATP depletion, and a subsequent failure in the homeostatic mechanisms that regulate cell

volume such as activation of the volume-regulated anion channel (VRAC), which requires ATP for its activity.

Importantly, our data only demonstrate that there should be a net influx of Na+ into the cell. The precise mechanism of cell death thereafter is speculative.

Testing whether low Na+ media could prevent cell death is an interesting idea. However, there are technical challenges to this experiment, including generating a Na-free media that still permits cell viability. We do not know if NMDG acts as a competitive blocker of Na+, so supplementing DMEM media with NMDG may not be sufficient to block Na+ influx into cultured HEK293 cells. However, this would be an interesting point to address in future studies.

Finally, and again this is just a question, not a request for an experiment, it would be interesting to know if zeel1 blocked the ability of peel1/pmpl1 to form channels in lipid bilayers. Zeel could be acting in lots of different ways. It could be altering association between the proteins, plasma membrane localization, or activity. These are not the topic of the paper, which is focused squarely on what is needed for peel1 to kill and how it kills. Nonetheless, the questions could be posed (see below).

Testing whether purified ZEEL-1 can block PEEL-1/PMPL-1 channel activity *in vitro* would be an interesting experiment. However, we have not successfully purified ZEEL-1 from bacteria. We hope to overcome this technical limitation in future work. We have added some speculation on the mechanism of ZEEL-1 antidote activity to the discussion section of the manuscript.

My one suggestion for additions to the paper is in the discussion. It is quite short. Since the paper overall is also not long, I think it would be useful if the authors were identify some of the outstanding questions related to interactions between peel1/pmpl1 and zeel1, and mechanisms of action, and ways they could be tested. In its current version the authors only spend one sentence suggesting that pmpl1 might activate the channel.

As suggested, we added several paragraphs to the discussion section, including speculation on the mechanism of antitoxicity, how PMPL-1 may interact directly or indirectly with PEEL-1, and how the planar lipid bilayer *in vitro* system can be used to determine more details of the ion channel.

In summary, this is a nice, focused paper, and the core results are exciting for those of us interested in toxin-antidote systems, how they work and how they evolved. The genetics is clear. The cellular localization studies are somewhat equivocal and primarily function to provide support for the plausibility of a mechanism of action at the plasma membrane. The electrophysiology and lipid bilayer work are not my areas of expertise, but seem to provide clear evidence that something interesting is happening in terms of changes in ionic conductance. While measurements of effects in whole cells can result from indirect effects, those observed with relatively pure combinations of proteins are compelling. The results presented herein set the stage for a number of future studies.

Full Revision

Bruce Hay

REFEREE CROSS-COMMENTS
I would agree with reviewer 3. While the experiments proposed by reviewer 2 are interesting, they fall outside the scope of this paper, which is focused on the identification of pmpl1 as an essential cofactor and the core data plausibly supporting the idea that some form of peel1 (with or without pmpl1) forms a channel.

As with my review and its comments on the discussion, a compromise would involve the authors noting that their work leaves open a number of exciting unanswered questions, including some related to biophysics of the channel and cell death, posed by reviewer 2.

We agree that a more detailed biophysical characterization of the ion channel is beyond the scope of our paper. As suggested, we have expanded the discussion, including speculation on the mechanism of cell death.

Reviewer #1 (Significance (Required)):

As noted above, this is an important paper because it begins to get at the mechanisms by which TA systems work to bias inheritance. Many such systems have been identified in diverse organisms, but we generally have little understanding of how they work or evolved. The cloning of pmpl1 as a necessary cofactor for peel1-dependent killing is an important breakthrough. The structural modeling, mutagenesis and electrophysiology and lipid reconstitution experiments provide important insights. At the same time it opens the door to many outstanding questions, which the authors could comment on a bit more.

the paper will be of general interest to those working in genetics, evolution, synthetic biology, structural biology and physiology.

Reviewer #2 (Evidence, reproducibility and clarity (Required)):

This study investigates the molecular mechanism of the PEEL-1/PMPL-1 toxin-antidote system in *Caenorhabditis elegans* and explores its toxicity reconstruction in HEK293T cells. The research reveals that PEEL-1, in cooperation with PMPL-1, forms a cation leak channel, leading to cell swelling and death, offering novel molecular insights into the toxicity mechanism in animal toxin-antidote systems. However, the experimental data remain overly simplistic in critical areas, requiring additional support to substantiate the conclusions. The main concerns are as follows:

1: While the data demonstrate the presence of a channel, the study lacks deeper characterization of the channel's dynamics and ion-specific current responses under different

conditions.
- (1) The lipid bilayer system, as an in vitro electrophysiological platform, has advantages in characterizing channel properties. The electrophysiological traces presented require further analysis; for example, this system could be used to verify the permeability to different ions and whether calcium permeability matches the whole-cell recordings. Additionally, single-channel data should be further analyzed.
- (2) The system should be utilized to confirm the loss-of-function mutations in channel function.

We appreciate that the reviewer agrees that the data in the paper support the conclusion that PEEL-1 and PMPL-1 form an ion channel. The proposed experiments to further characterize the biophysical details of the ion channel would be a great next step. However, we think that these experiments are beyond the scope of this manuscript. The goals of this study were to determine whether PEEL-1 required additional factors for toxicity, and to characterize the mechanism of toxicity at the cellular and molecular level. We have accomplished these goals by identifying PMPL-1 as being both necessary and sufficient for PEEL-1 mediated toxicity, shown how PEEL-1 and PMPL-1 coexpression is toxic to cells, and uncovered a plausible molecular role for PEEL-1 and PMPL-1 as components of a new cation channel. From our electrophysiology data, we can say that there is not much, if any, intrinsic voltage-dependence nor gating kinetics associated with PEEL-1/PMPL-1 channels. These channels appear to operate as classic non-selective monovalent cation "leak" channels. Determining the biophysical properties of the channel at a deeper level would be interesting, but is not essential for the major conclusions of the paper. We would argue that the electrophysiological data we do present are internally consistent and sufficient to support the conclusions we draw.

Further work analyzing the specific biophysical properties of the ion channel will be technically challenging and time consuming. To analyze single-channel data, we would first need to verify that we have a single channel present in the bilayer. This may be especially difficult if the ion channel has multiple oligomeric states with different properties, which is suggested by our preliminary data. Often, entire papers are devoted to just characterizing the ion selectivity of an ion channel, even of ion channel families that have been studied for decades. We are limited in this work since this ion channel is novel and has no similarity to known ion channels. This also means we have no idea of what single-channel conductances we might be expecting.

Testing ion selectivity *in vitro* and assaying mutant proteins for changes in ion selectivity are important next steps. Unfortunately, our *in vitro* system is very low throughput and purifying these transmembrane proteins is difficult and labor intensive. For each mutant tested, a new protein will need to be expressed and purified. Additionally, we cannot precisely control the stoichiometry of the proteins, nor the orientation in which the proteins are inserted in the bilayer (N-terminal vs C-terminal facing the trans compartment). We are working on a different system where we can assay ion channel activity in liposomes rather than in planar lipid bilayers, but this method also has technical issues we are still trying to overcome.

- (3) Please use this system to investigate the detailed interaction mechanisms between PEEL-1 and PMPL-1.

We are unclear exactly what the reviewer has in mind in terms of using the planar lipid bilayer system to investigate PEEL-1 and PMPL-1 interaction mechanisms. One possibility is to use point mutants which specifically disrupt potential protein-protein interactions between PEEL-1 and PMPL-1. However, we have not yet confirmed a direct interaction biochemically and do not know which residues might be responsible for such an interaction. The lipid bilayer assay is too low-throughput to reasonably test many point mutants in PEEL-1 and PMPL-1. It is also possibly that the interaction between PEEL-1 and PMPL-1 is indirect, and thus would require more work to determine how PMPL-1 may modify the local lipid environment. Determining the interaction mechanism between PEEL-1 and PMPI-1 is not necessary for the conclusions we draw in the paper, so we consider these experiments to be beyond the scope of this work.

2. While the structural prediction suggests that the amphipathic α-helix of PEEL-1 may form the channel lining, this hypothesis lacks validation through mutagenesis or structural biology approaches.

We agree that this hypothesis has not been confirmed in our study and have been careful in our writing to make this clear to the reader. Our mutagenesis studies in Figures 4 and S10 indicate that both the presence and the amphipathic property of the helix are important for toxicity. We only speculate that it serves as the lining of a channel pore based on other channel-forming proteins, but also mention other alternative possibilities: "These data suggest that the amphipathic property of the PEEL-1 amphipathic helix is critical for toxicity, supporting its potential role as the lining of a channel. However, we cannot rule-out alternative explanations for the importance of the AH, including roles in protein-protein interaction or protein stability." It would be valuable to perform electrophysiological experiments on mutants in the amphipathic helix to determine if they change properties of the channel such as ion selectivity or conductance, but such experiments will be an enormous amount of work. We consider these experiments to be beyond the scope of this paper.

Structural biology approaches (e.g. cryo-EM or X-ray crystallography) may be complicated by the possibility that oligomeric states may be short-lived or that there may be variability in oligomeric states. Given that there are multiple truncation products in our purified PEEL-1 protein preps and yields are relatively low, any attempt to determine its structure would first require optimization of the protein expression and purification protocol. While structural biology would expand our understanding of the channel, such experiments are very laborious and are not necessary for the main conclusions of the manuscript. Thus, we consider them to be beyond the scope of the current paper.

3. Although the co-expression of PEEL-1 and PMPL-1 results in cell death, the interaction mechanism between these two proteins is unclear. Do they have binding affinity, or are they in a

downstream regulatory relationship? The co-localization data is also confusing-if these proteins do not co-localize, how do they function together?

We agree that the interaction mechanism between PEEL-1 and PMPL-1 is unclear. We have added to the discussion section to describe some possibilities.

We have attempted to test for interactions between PEEL-1 and PMPL-1 *in vitro* but results have been difficult to interpret. *In vitro* experiments are complicated by the fact that these transmembrane proteins must be purified in detergent to make them soluble. These detergents may disrupt the protein-protein interactions we are attempting to detect. In general, *in vitro* experiments with transmembrane proteins are difficult. We have attempted several other experiments to test whether PEEL-1 and PMPL-1 interact, including co-immunoprecipitation, yeast 2-hybrid, and bimolecular fluorescence complementation. Results from these experiments have been unclear. Therefore, we can only speculate on how or whether PEEL-1 and PMPL-1 interact.

In terms of co-localization, we apologize for not being clear in our manuscript. We do see PEEL-1 and PMPL-1 co-localize at the ER and plasma membrane, consistent with the idea that they function together. This is most apparent in Figures 3D and S12A. Our ER-retention experiments in Figure 2E indicate that PEEL-1 and PMPL-1 likely act together at the plasma membrane. We have clarified PEEL-1 and PMPL-1 co-localization in our manuscript. The text now reads:

"PEEL-1 and PMPL-1 localize to several membrane-bound compartments in HEK293T cells and *C. elegans*. Both proteins localize to the endoplasmic reticulum (ER), nuclear envelope-associated ER, and plasma membrane (PM) (Fig. 2C-D and Fig. S6B-C). Additionally, PMPL-1 localizes to lipid droplets (Fig. 2D and Fig. S6A), consistent with the predicted monotopic topology. Although PEEL-1::eGFP is not easily detectable on the PM of transfected HEK293T cells, stable expression of PEEL-1 in HEK293 cells or in *pmpl-1* knock-out worms shows clear PM localization (Fig. S6B-C). This also suggests that PEEL-1's trafficking to the plasma membrane does not require PMPL-1. Since PEEL-1 and PMPL-1 co-localize on the ER and PM, we hypothesized that they act together at one of these locations to induce toxicity."

4. I encourage the authors to explore and discuss the possible mechanisms of cell death mediated by PEEL-1/PMPL-1.

As suggested, we have expanded our discussion, speculating on more precise mechanisms of how PEEL-1 and PMPL-1 may cause cell death.

Reviewer #2 (Significance (Required)):

This study investigates the molecular mechanism of the PEEL-1/PMPL-1 toxin-antidote system in *Caenorhabditis elegans* and explores its toxicity reconstruction in HEK293T cells. The research reveals that PEEL-1, in cooperation with PMPL-1, forms a cation leak channel, leading to cell swelling and death, offering novel molecular insights into the toxicity mechanism in animal toxin-antidote systems. However, the experimental data remain overly simplistic in

critical areas, requiring additional support to substantiate the conclusions.

Reviewer #3 (Evidence, reproducibility and clarity (Required)):

This paper describes the mechanism of action of a toxin-anti-toxin protein pair in C. elegans and a mechanistically critical interaction with an endogenous cellular component. The paper demonstreates that the PEEL-1 toxin works with PMPL-1 protein to promote cell swelling and death. Expression of both proteins is sufficient to induce death in a heterlogous system, and this can be inhibited by the anti-toxin. The paper uses a panoply of methods to look at the activity of the various components, including biochemisty, membrane conductance and genetic studies.

I think the paper is a remarkable tour-de-force, and I have no scientific issues. I believe the authors have done an outstanding job characterizing this system.

We appreciate the reviewer's positive comments. Thank you so much.

REFEREE CROSS-COMMENTS
I have read the comments of the two other reviewers. While I agree that experiments suggested by reviewer 2 are interesting and will yield further understanding of the peel/zero system, I believe they are largely outside the scope of the current manuscript, and better suited for a follow up manuscript. In general, I believe that additional experiments should be limited to correcting scientific problems in the manuscript.

We agree that a more detailed biophysical characterization of the ion channel is beyond the scope of our paper, and is not critical for the major conclusions of our paper.

Reviewer #3 (Significance (Required)):

Toxin-antitoxin systems are prevalent in bacteria, but also exist in eukaryotes. They can tell us about gene evolution and natural biological warfare, and can be used to develop various technologies for cell ablation. The studies here characterize a C. elegans T-A system in depth. Although there are of course more questions to answer- e.g. regarding the reason for the PEEL-1 and PMPL-1 interactions, the breadth and depth of the work described here is impressive.
