# Supplementary material for: An animal toxin-antidote system kills cells by creating a novel cation channel
Source: PLoS Biol. 2025 May 27;23(5):e3003182. doi: 10.1371/journal.pbio.3003182 (PMC12136403; doi:10.1371/journal.pbio.3003182)
Supplement: Attachment — Submitted filename: response to reviews.docx [file pbio.3003182.s026.docx]

Point-by-point response to Reviewers

REVIEWS:


Reviewer #1 (Bruce A Hay, signs review): I appreciate the author comments and the changes to the discussion.

My one minor comment in rereading the manuscript is in the beginning of the results. The authors say "Endogenous, sperm-delivered PEEL-1 is also
suppressed by pmpl-1 mutations (Fig. 1C), but not via a paternal-effect"

I think they mean "Killing by endogenous..."

**We have made this change.**

Reviewer #2: I dont have further comments on the manuscript, which is appropriate to be published as a short discovery article.


Reviewer #3: The authors have addressed all concerns. I have no further issues.


Reviewer #4: I read the discussion and the revised manuscript. Unfortunately I m not an expert in the biological part so I will not comment on that. Concerning the electrophysiology I consider the data and the conclusion sufficient. Obviously a detailed study can be made but this would be study by its own.
The cellular work shows pore forming activity and reconstitution in planar lipid bilayer a distribution of discrete conductances. As the overall study sounds very interesting I suggest to publish as it is.

**We thank the original three reviewers for suggesting publication of the manuscript as is. We also thank the fourth reviewer who was added for their expertise in planar lipid bilayer experiments. We agree that a detailed study of the properties of this ion channel would be its own separate study. We are delighted that this additional reviewer finds our study interesting and data/conclusions sufficient to publish as is.**

COMMENTS FROM THE ACADEMIC EDITOR

After carefully going over the manuscript again, I still have some concerns about the bilayer experiments. I think the authors should address these in their manuscript by providing appropriate control experiments and a better characterization of their data to provide stronger evidence that the observed currents in planar bilayers have equivalent properties to the currents measured in HEK-293 cells.

**We note that there is no necessary expectation that the *in vitro* currents measured in planar lipid bilayers will match the currents measured in cells, since many variables can affect *in vitro* behaviors, including lipid composition, membrane fluidity, and broader membrane composition. To delve into such issues properly, optimized conditions would need to be found by systematically varying many parameters, and this would require a significant amount of work. We feel that such experiments are outside the goals and scope of our manuscript. In this manuscript, what we can conclude with confidence is that these bilayer events are unitary events, requiring both PMPL and PEEL proteins, and are not an artifact of the expression system or purification.**

In light of the positive remarks of all four reviewers I regard the chance that the paper will be accepted as very high. I also do not think that it should be sent for re-review and that it could even be published without the bilayer data in case these turn out to be inconclusive.

The questions I have concerning protein expression, purification, reconstitution and bilayer recordings are as follows:

Protein expression and purification:

It was not confirmed that E. coli would be an appropriate overexpression system for both proteins, particularly since eukaryotic membrane proteins are only in exceptional cases functionally overexpressed in prokaryotic cells.

**Though there are challenges with expressing eukaryotic membrane proteins in bacteria, there are numerous studies that have done so successfully, including expression of a number of eukaryotic ion channels (Kesidis et al. 2020, PMID: 32534131). Our proteins are quite small (PEEL-1 = 174 residues and PMPL-1 = 59 residues) which are historically more well-behaved than larger membrane proteins for purification from bacteria. It is difficult to test whether E. coli is a suitable expression system since there is no established functional assay for PEEL-1 and PMPL-1. The only way to know if a system is an appropriate expression system is if it produces functional protein, but our only way to assay for PEEL-1 and PMPL-1 function is to use the planar lipid bilayer system. Thus, it is a bit of a circular argument to say that this system has not been confirmed as being appropriate.**

I also noticed that the authors extract their proteins after dounce-homogenization, which I do not regard as efficient in disrupting bacterial cells, followed by extraction in buffer containing 2% of the detergent Octyl-Glucoside. I thus wonder which fraction of the totally overexpressed proteins is captured in that way.

**The use of Dounce homogenization, combined with lysozyme and 2% beta-octyl-glucoside is indeed a mild lysis procedure. We chose this method of lysis to increase the likelihood of preserving protein function, albeit at the cost of yield. The yields of MBP-tagged PMPL-1 were quite high using this method, indicating that we are successfully purifying proteins. Yields from MBP-PEEL-1 were lower, but this is likely due to the toxicity of the full-length protein. Regardless of the efficiency of lysis, we argue that what matters most is whether the purified proteins are functional. Our planar lipid bilayer assays suggest that these purifications indeed produce functional protein, since neither PEEL-1 nor PMPL-1 alone permits ion flux through the bilayer, but when combined, they produce a distribution of discrete conductances.**

I also wonder why the authors record the absorbance at 260 and not 280 nm to estimate protein yields.

**This was a typo that we have corrected. We measured protein concentration at 280 nm.**

It would be helpful, if the authors would characterize their purified protein by size exclusion chromatography to estimate the oligomeric organization and dispersity of the sample and separate the fraction of the protein with appropriate oligomeric state from aggregates and incompletely assembled populations.

**We attempted to characterize the oligomeric states of our purified PEEL-1 and PMPL-1 proteins using mass photometry. We found that PEEL-1 was mostly monomeric, while PMPL-1 was a mix of oligomers. These experiments also showed that there was little aggregated protein in our samples. We agree that further characterization of the oligomeric states of these proteins would be informative, but we feel that such experiments are outside the scope of the current manuscript and more suited for a detailed biophysical and structural characterization of the channel. Although performing size exclusion chromatography of our protein preps sounds simple enough, since we don’t know what the appropriate oligomeric state is, we would need to test each fraction in planar lipid bilayers for function, which becomes a lot of work given the low-throughput nature of these experiments. Also, chromatography becomes very expensive because of the detergent included in the buffers. Additionally, any characterization of the purified protein (including oligomerization state) could be completely nullified because the proteins are incorporated into liposomes prior to use for planar lipid bilayer experiments, and it is possible that the oligomeric state of the protein in detergent will not be the same as the oligomeric state in liposomes.**

I think that this information is very important for several reasons:
1. Octyl glucoside is a comparably harsh detergent that is compatible with few stable membrane proteins (typically of prokaryotic origin) but that leads to aggregation in other cases.
2. The protein is expressed as MBP fusion, which tends to increase the solubility of misfolded membrane proteins and which was not cleaved off after purification.

**These two points are both concerned with the possibility that are our purified proteins are aggregates. Several observations argue against aggregation being a significant problem for our experiments. First, we do not observe aggregates in mass photometry experiments using purified PEEL-1 and PMPL-1. Second and most importantly, even if there are some aggregates in our protein preps (which we acknowledge is possible in our manuscript), it is highly unlikely that aggregation causes the ion-channel activity observed in planar lipid bilayer experiments given that this activity is seen only when PEEL-1 and PMPL-1 are combined, but not when they are tested individually. These PEEL-1 only and PMPL-1 only controls refute many of the concerns brought up by the Academic Editor that the observed activity could be due to some artifact of our protein preps.**

**β-octyl-glucoside was selected as a detergent after a detergent screen of four different detergents. Of these detergents, we found that β-octyl-glucoside provided the most monomeric PEEL-1 and PMPL-1 protein, analyzed by mass photometry. MBP was used as a tag since it improved the solubility of our expressed proteins. However, MBP is not known to have any channel activity on its own, which is corroborated by our PEEL-1 and PMPL-1 individual controls.**

3. Size exclusion chromatography could provide evidence for the proposed pentameric organization.

**As mentioned above, we think that a detailed characterization of oligomeric states is outside the scope of the current manuscript. We also point out the problem that even were we to define specific oligomeric states of our purified proteins assembled in detergent, these states may not be the same as those in liposomes (both of which may differ from native states in cells). We acknowledge in the text that the pentameric state is merely a prediction. But we do note that this model was able to guide a mutagenesis experiment that gave a positive result: the negatively charged aspartate residue that may form a ring of charge at the extracellular mouth of the putative pore was required for PEEL-1 activity in cells.**

Bilayer recordings:

The authors should provide better evidence that the currents measured in their bilayer experiments have similar selectivity and current-voltage relationships as macroscopic currents. So far, the currents were only recorded at strongly positive voltages (180 mV) and at very high salt concentration (600 mM NaCl) and I do not know why the authors have decided for such drastic conditions.

**These voltages were chosen because they are standard for the nanopore experiments that our collaborators perform. We did try smaller voltages, but we found that this higher voltage (for unknown reasons) gave us more robust liposome fusion. The high salt concentration was chosen for liposome fusion, since the higher salt outside of the liposomes encourages them to destabilize once they make contact with the planar lipid bilayer, promoting liposome fusion and delivery of our proteins to the bilayer. There is room for optimization in salt and lipid composition to perform the assay with physiological voltages, but this assay is very low-throughput and optimization would require several months of experimentation. Additionally, we do not claim that the currents we observe *in vitro* are identical to the currents produced in cells. This assay simply shows that when PEEL-1 and PMPL-1 are combined, they produce ion flux through a bilayer.**

**We agree that testing ion selectivity and voltage-dependence in *in vitro* experiments are important next steps in characterizing this channel, but are more suited to a separate study characterizing channel properties in depth. And as mentioned above, given the many differences between planar lipid bilayers and cells, one would not expect that the channels necessarily exhibit exactly the same properties.**

I also wonder why these currents show the observed strong fluctuations and why they did not stabilize over time, particularly since macroscopic currents in Hek cells did not show a pronounced time dependence.

**This could be due to many possible reasons, including differences in the lipid composition of planar bilayers or the presence of truncated proteins in these experiments. It is also possible that single-channel fluctuations seen in planar lipid bilayer experiments may be masked in HEK293 cells where we record whole-cell currents contributed by a population of channels. To answer this question would require a much more in-depth study of channel properties.**

It would also be interesting to know whether the authors have observed stable macroscopic currents in their experiments that would facilitate the recording of meaningful current voltage relationships. If the spikes observed in Fig. 16 B, C would indeed correspond to the fusion of single vesicles with the bilayer, I would expect a constant current increase due to the incorporation of an increasing number of channels also in the recordings displayed in Fig. 16 D, E.

**We did try this, but we could not obtain meaningful current/voltage relationships because of the variability of channel activity.**

Comparisons of current voltage relationships and recordings in asymmetric ionic conditions would help to clarify these issues.

**We agree these would be interesting experiments, but again, they would be a significant amount of work in this low-throughput system, and outside the scope of the current manuscript. Our manuscript establishes that PEEL-1 and PMPL-1 together form a channel, but does not claim to characterize all of the properties of this channel. Often, entire papers are devoted to just characterizing the ion selectivity of an ion channel, even of ion channel families that have been studied for decades. We are limited in this work since this ion channel is novel and has no similarity to known ion channels.**

I regard these control experiments as essential since planar bilayer experiments are prone to artifacts and unfolded proteins have previously been shown to induce single-channel like properties.

**We would like to emphasize again that our PEEL-1 only and PMPL-1 only control experiments argue strongly against artifactual ion-channel activity due to misfolded proteins in these preps. We observe ion channel activity only when we combine PEEL-1 and PMPL-1 proteins. It is unclear how such artifacts could lead to these results. However, we have added a sentence to the Discussion stating explicitly that “These planar lipid bilayer experiments are preliminary and further work will be required to fully characterize the ion channel activity.”**
